# Simultaneous Determination of the Size and Shape of Single α-Synuclein Oligomers in Solution

**DOI:** 10.1021/acsnano.3c01393

**Published:** 2023-06-16

**Authors:** Saurabh Awasthi, Cuifeng Ying, Jiali Li, Michael Mayer

**Affiliations:** † Adolphe Merkle Institute, University of Fribourg, Chemin des Verdiers 4, CH-1700 Fribourg, Switzerland; ‡ University of Arkansas, Fayetteville, Arkansas 72701, United States

**Keywords:** amyloid oligomers, α-synuclein, aggregation, nanopore, resistive pulse sensing

## Abstract

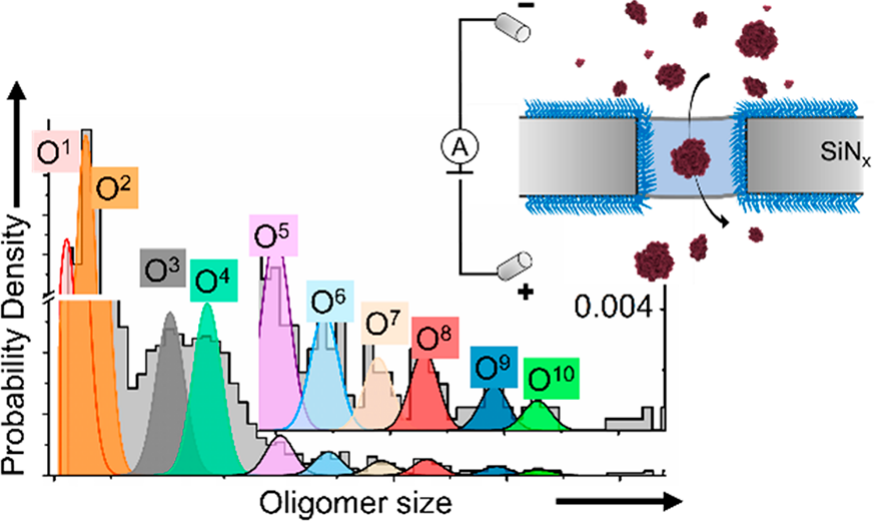

Soluble oligomers of amyloid-forming proteins are implicated as toxic species in the context of several neurodegenerative diseases. Since the size and shape of these oligomers influence their toxicity, their biophysical characterization is essential for a better understanding of the structure–toxicity relationship. Amyloid oligomers are difficult to characterize by conventional approaches due to their heterogeneity in size and shape, their dynamic aggregation process, and their low abundance. This work demonstrates that resistive pulse measurements using polymer-coated solid-state nanopores enable single-particle-level characterization of the size and shape of individual αSyn oligomers in solution within minutes. A comparison of the resulting size distribution with single-particle analysis by transmission electron microscopy and mass photometry reveals good agreement with superior resolution by nanopore-based characterization. Moreover, nanopore-based analysis has the capability to combine rapid size analysis with an approximation of the oligomer shape. Applying this shape approximation to putatively toxic oligomeric species that range in size from 18 ± 7 aggregated monomers (10S) to 29 ± 10 aggregated monomers (15S) and in concentration from picomolar to nanomolar revealed oligomer shapes that agree well with previous estimates by cryo-EM with the added advantage that nanopore-based analysis occurs rapidly, in solution, and has the potential to become a widely accessible technique.

Neurodegenerative diseases characterized by misfolding and aggregation of α-synuclein (αSyn) protein (14.5 kDa) are collectively termed synucleinopathies.^1−4^ The most common types of synucleinopathies are Parkinson’s disease (PD), dementia with Lewy bodies (DLB), and multiple system atrophy (MSA).^3^ Oligomers of αSyn are neurotoxic species and implicated in the pathology of Parkinson’s disease.^5−9^ Colla et al. showed that αSyn accumulates within the endoplasmic reticulum or in microsomes forming toxic oligomers in transgenic mouse brain, leading to α-synucleinopathy.^10^ Prots et al. demonstrated that αSyn oligomers disrupt axonal integrity in human neurons and that increased αSyn oligomerization by expressing oligomer-forming mutants (E46K and E57K) of αSyn resulted in impaired axonal transport of mitochondria.^11^

Although most soluble oligomeric species of αSyn are toxic,^11^ the molecular basis of toxicity remains to be established.^12^ Therefore, the biophysical characterization of αSyn oligomers is essential for understanding their pathology and for developing therapeutic interventions. Characterization and quantification of αSyn oligomers, remains, however, challenging with established approaches due to the heterogeneity of oligomeric species with regard to their size, shape, and low abundance with concentrations ranging from picomolar (pM; 10^–12^ mol L^–1^) to nanomolar (nM; 10^–9^ mol L^–1^).^13,14^ Van Steenoven et al., using enzyme-linked immunosorbent assays, reported cerebrospinal fluid (CSF) levels of oligomeric αSyn in samples from patients with PD to be as low as 120 ± 49 and 72 ± 37 pg/mL for healthy control subjects.^15^ One reported mechanism of toxicity of αSyn oligomers involves interactions with lipids and formation of pores in phospholipid membranes.^16−18^ The physical properties of αSyn oligomers such as oligomer size or conformation/shape are key determinants of their pore-forming activity and toxicity.^18−23^ Specific sizes (i.e., oligomers with a certain number of monomers) or specific shapes (such as annular or disc-shaped) of oligomers have been associated with neurotoxicity in vivo.^9,18,21^ Recently, Kiechle et al. used in vivo protein complementation to identify presynaptic oligomerization and age-dependent accumulation of potentially toxic αSyn oligomers in a specific size range (8–16-mer).^24^ Also, prior studies have identified and demonstrated neurotoxic αSyn oligomers composed of ∼30 monomers,^25^ >30 monomers, and 10–40 monomers.^21,26^

Characterization methods based on ensemble analysis, such as size exclusion chromatography (SEC), analytical ultracentrifugation (AUC), small-angle X-ray scattering (SAXS), dynamic light scattering (DLS), and gel electrophoresis, fail to capture the entire range of heterogeneity in size with good resolution, and they either cannot determine oligomer shape or struggle to do so in heterogeneous mixtures. In addition, the need for fluorophore labeling, cross-linking, or preparation of dry samples of some existing methods may result in undesirable alterations in the physical and biochemical properties of amyloid oligomers.^27,28^ Recently, Arter et al. used microfluidic free-flow electrophoresis as a solution-based approach to fractionate stable αSyn oligomer ensembles for achieving structural, kinetic, and immunological characterization in addition to the measurement of average oligomer ζ-potential of each fraction.^29^ Alternative, single-molecule methods have employed fluorescence methods such as single-molecule Förster resonance energy transfer (smFRET),^22^ single-molecule photobleaching to characterize αSyn oligomers in vitro with respect to oligomer size,^30^ and single-molecule fluorescence burst measurements to detect the formation of oligomeric aggregates.^31,32^ The goal of the work presented here was to address the challenge of analyzing heterogeneous oligomer samples by establishing an accessible and practical method for characterizing αSyn oligomers on a single oligomer level, rapidly and in solution.

An elevated level of αSyn oligomers in CSF or plasma is associated with the onset and progression of neurodegenerative synucleinopathies;^33,34^ therefore, αSyn oligomers are promising biomarker candidates.^33,35^ Commonly used immunoassays to quantify amyloid oligomers such as enzyme-linked immunosorbent assay (ELISA),^36,37^ single-molecule array (SIMOA) assays,^38,39^ and surface-based fluorescence intensity distribution analysis (sFIDA)^40^ require the use of high-affinity antibodies and typically fail to discriminate oligomers from monomers and fibrils. Moreover, a recent study by Kumar et al. tested 16 so-called “conformation-specific αSyn antibodies”, and none of these antibodies showed specificity for any particular aggregate size of αSyn.^38^ Given the current challenges associated with immunological assays for oligomer quantification, an urgent need exists for an antibody-free, sensitive approach that can determine oligomer size, abundance, and ideally shape.

We propose that one attractive approach to address this need is to monitor aggregation using nanopores.^41−49^ This approach takes advantage of a transient increase in electrical resistance when particles move through electrolyte-filled nanopores, a mode of detection that does not require labeling of particles.^41,42,44,45,49,50^ Three types of nanopores are commonly employed for the detection of oligomers of amyloid-forming proteins: First are protein nanopores (so-called biological nanopores), which have the advantage that they can be produced with exquisite reproducibility and that they typically do not clog during measurements, while having the disadvantage that, thus far, their pore diameters are typically smaller than 5 nm, limiting them to the detection of monomers or the smallest oligomers of amyloid-forming proteins.^41,51^ Second, nanopores at the tip of glass capillaries or in polymer films have the advantage that they can be readily produced, while their conical and elongated pore shape hinders the estimation of particle shape.^44,52,53^ And third, nanopores in solid-state SiNx membranes have the advantage that they can be fabricated with a large range in diameters in order to adjust it to the size of oligomers of interest.^42,54^ Possible disadvantages of these pores are that their fabrication is typically a serial process, that it requires specialized equipment, that their diameters tend to grow during experiments due to slow etching in aqueous electrolyte, and that coatings must be employed to minimize nonspecific interactions of proteins with the walls of the nanopore.^48,55−57^ So far, most studies using nanopores in the context of analyzing amyloid oligomers focused either on the detection of oligomers or on monitoring the aggregation process over time; typically these studies could not determine the size of amyloid oligomers, and even fewer studies were able to interrogate oligomer shapes.^42,44,49−51^ For reviews of progress in this field, please see Houghtaling et al.,^49^ Meyer et al.,^52^ and Chen et al.,^58^ as well as recent reports.^43,44,46,47,59,60^ In addition, nanopore-based methods are also being used to follow ligand-induced conformational changes in proteins^61^ and to study the enzymatic process^62^ on a single-molecule level.^63,64^

Here, we apply nanopore-based resistive pulse recordings to the single-particle characterization of oligomers of αSyn and reveal the size and shape of αSyn oligomers. We demonstrate that the size distribution of αSyn oligomers contains 10 subpopulations of oligomers with a distinct and stable number of aggregated monomers. Supported by TEM-based single-particle imaging and mass photometry analysis, these stable oligomer species start with tetramers and 12-mers and then contain multiples of approximately 12 monomers, including 24-mers, 48-mers, 60-mers, and 84-mers. Moreover, the size and shape analysis of individual oligomers makes it possible to detect and quantify oligomers of specific size, which have previously been identified as “toxic oligomers” such as 10S and 15S,^65^ and to reveal the distribution of the shape of oligomers with these sizes. In this way, the abundance of a subpopulation with putatively toxic size and putatively toxic shape (such as prolate-shaped or tubular oligomers^18,21^ that possess pore-forming activity) may be determined.

## Results/Discussion

### Analysis of the Size of α-Synuclein Oligomers at the Single-Particle Level

**Figure 1:**
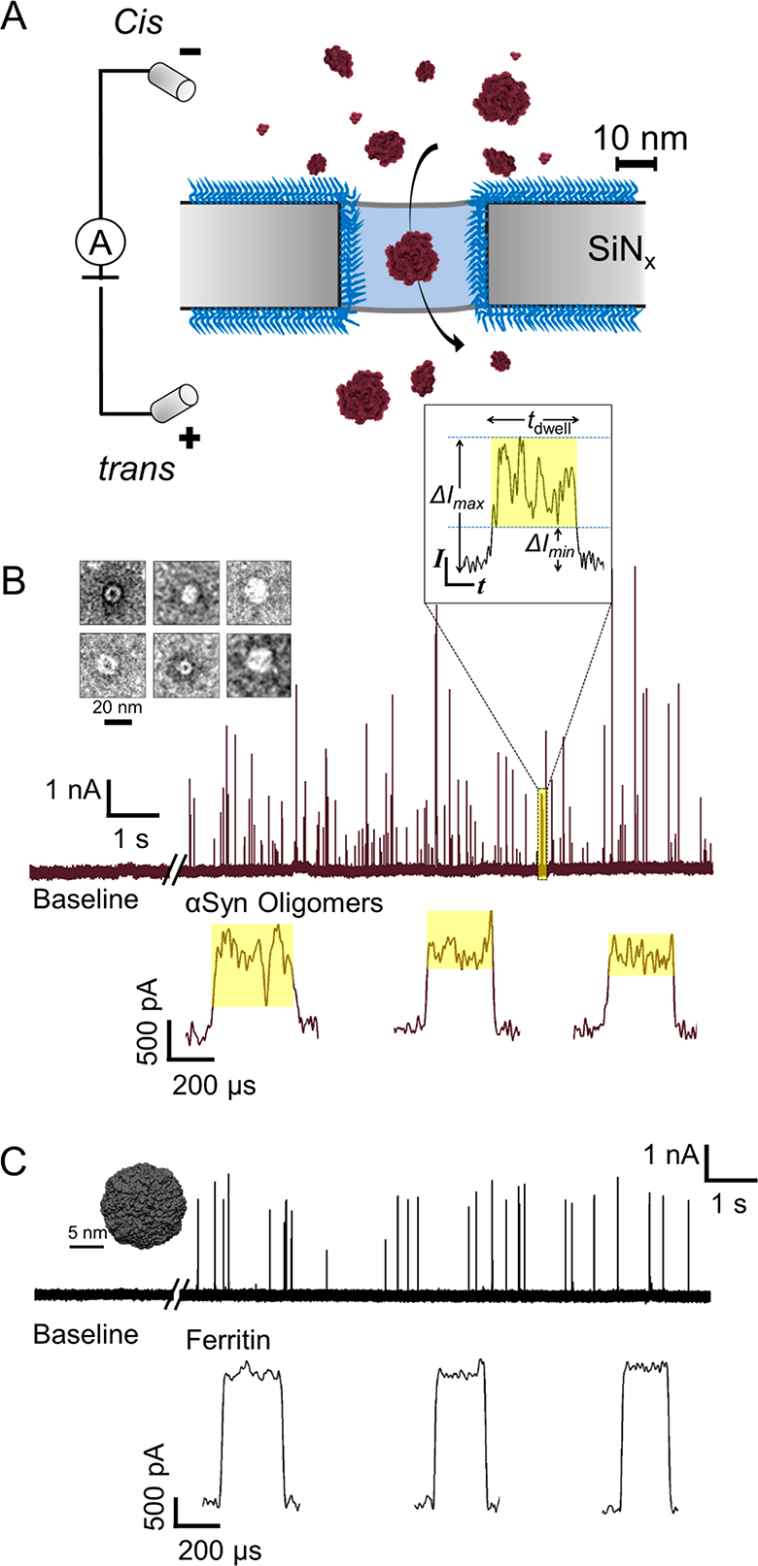
Nanopore-based, single-particle characterization of α-synuclein oligomers in solution. (A) Schematic illustration of the experimental setup to measure resistive pulses due to the translocation of individual αSyn oligomers through a polymer-coated nanopore (PAcrAm-*g*-PMOXA polymer,^57^ blue). (B) Original current recordings, before and after adding αSyn oligomers, show resistive pulses due to the translocation of αSyn oligomers as upward spikes (nanopore diameter = 25 nm, voltage = −100 mV applied to the top electrode, mean current = 23.1 nA). The TEM image in the inset shows individual αSyn oligomers from the sample used for the nanopore experiments. Characteristic translocation events from the passage of individual αSyn oligomers through the nanopore illustrate the dwell time (*t*_d_), minimum current blockade (Δ*I*_min_), and maximum current blockade (Δ*I*_max_) that arise from the rotational dynamics of nonspherical oligomers translocating through the electric field inside the nanopore. The magnitude of current blockade Δ*I* is proportional to the particle volume, while the intra-event current modulations (shaded in yellow) contain information about the shape (*m*) and orientation of each oligomer in solution.^60,66^ Three additional examples of translocation events (*t*_d_ > 150 μs) from the passage of individual αSyn oligomers through a nanopore are shown below. (C) Original current recordings before and after adding the perfectly spherical protein ferritin (PDB ID: 6TSF) show resistive pulses as upward spikes due to single protein translocations (nanopore diameter = 25 nm, voltage = −100 mV applied to the top electrode, mean current = 22.8 nA). Note that the three examples of translocation events that are shown in detail (*t*_d_ > 150 μs) from the passage of ferritin through the same nanopore result in square-shaped resistive pulses with small intra-event current modulations. For this work, we used nanopores that resulted in such square-shaped resistive pulses for ferritin translocations, indicating that these pores correctly indicate small current modulations from rotations of a perfectly spherical protein. In this case, the modulations are similar in amplitude to the baseline noise, as reported before.^60,66^

Figure 1A illustrates the experimental setup for resistive pulse measurements using solid-state nanopores. A single pore with a diameter of 25–56 nm in a thin insulating silicon nitride (SiN_*x*_) membrane separates two electrolyte compartments. A high-gain, low-noise patch-clamp amplifier applies a potential difference (±0.1 V) between these compartments and monitors the ionic current through the nanopore. Passage of individual αSyn oligomers through the nanopore displaces the conducting electrolyte, resulting in characteristic resistive pulses. These resistive pulses contain information about the physical properties of the translocating particles, including their volume (i.e., size “Λ”) and shape (i.e., length-to-diameter ratio “*m*” of a corresponding ellipsoidal model) (see Figure 1B).^60^ The dwell time during which a protein resides in the pore, *t*_d_, is influenced by its electrophoretic mobility and hence its net charge. For the work presented here we employed nanopores for which individual translocation events as a result of the passage of the perfectly spherical protein ferritin resulted in square-shaped resistive pulses with small intra-event current modulations (see Figure 1C). All the nanopores used for this work were preselected to result in such square-shaped resistive pulses for ferritin translocations; these pores correctly detect small current modulations from rotations of a perfectly spherical protein.^60,66^

**Figure 2:**
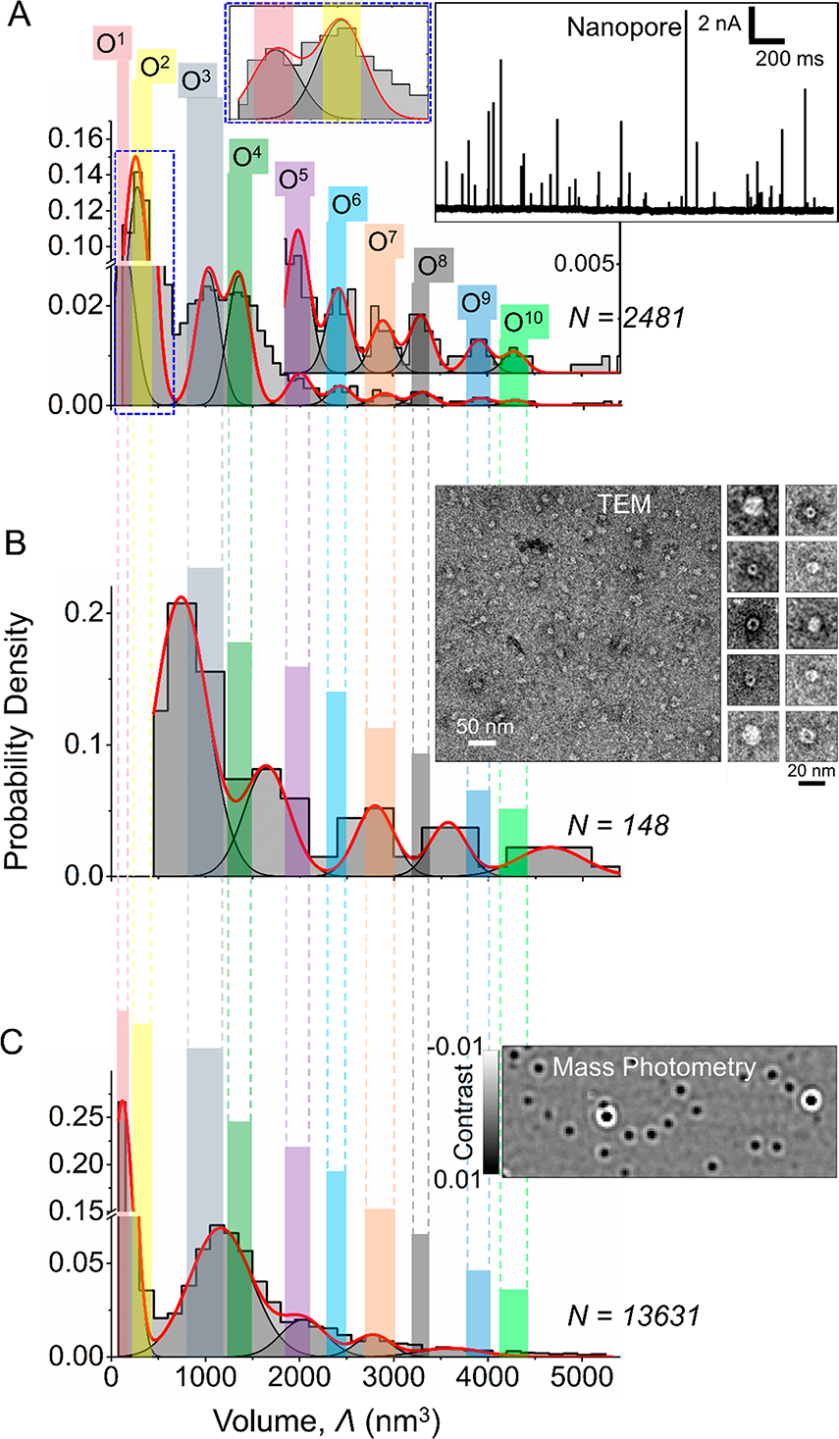
Nanopores enable analysis of oligomer size distributions with high resolution. (A) Size distribution of αSyn oligomers determined using nanopore recordings. Differently sized oligomer subpopulations identified using the nanopore-based approach are marked as O^1^ to O^10^ and range in size from a (4 ± 3)-mer to (122 ± 5)-mer. The inset with a blue dashed outline shows the first two oligomer peaks. The other inset shows original current recordings through a nanopore with resistive pulses due to the translocation of single αSyn oligomers as upward spikes. We used three different nanopores with diameters ranging from 25 to 56 nm to capture the wide distribution of αSyn oligomer sizes (Supplementary Figure S4). All three pores were preselected for accurate volume and shape quantification of the spherical protein ferritin (see Materials/Methods). (B) Size distribution of αSyn oligomers determined using TEM. The inset shows a TEM micrograph of αSyn oligomers used for the analysis. The resolution is limited because only two-dimensional images can be obtained and the particle volume was estimated considering the long axis as the particle diameter of the 2D projection (C) Size distribution of αSyn oligomers determined using single-particle mass analysis by mass photometry. The inset shows a differential interferometric scattering image of αSyn oligomers. For comparison, the shaded regions in different colors show the presence and absence of different oligomer populations identified by nanopores, TEM, and mass photometry. Since mass photometry is calibrated to reveal the molecular weight of each particle, we converted the molecular weight to particle volume using the molecular weight of αSyn monomers (14.5 kDa) and assuming a monomer volume of 35 nm^3^.

Figure 1B shows original current traces of electrical recordings through nanopores, featuring translocation events of individual oligomers as resistive upward spikes (i.e., reductions in the amplitude of the negative baseline current). In order to evaluate the benefits of nanopore-based oligomer characterization on a single-particle level, we employed samples of αSyn oligomers obtained from ND Biosciences, Switzerland, which were prepared as explained by Kumar et al.^67^ To determine the size distribution of these samples, we recorded thousands of individual translocation events for αSyn oligomers. We classified αSyn oligomers based on their size in terms of the number of monomers they contain. To this end, we used the volume of monomeric αSyn protein, which we estimated to be 35 nm^3^ using nanopore experiments (see Supplementary Note 1 and Supplementary Figure S1), in good agreement with earlier reports of αSyn monomer volume (∼30 nm^3^) determined by SAXS.^68^ Oligomer sizes estimated by nanopores range from a dimer to large-sized oligomers consisting of ∼150 monomers (Figure 2), again in good agreement with the sizes of αSyn oligomers reported earlier.^21,22,69^Figure 2A shows that single-oligomer analysis of these samples using nanopores revealed 10 maxima in the size distribution corresponding to 10 different sizes within the oligomer population. We labeled different-sized subpopulations of oligomers as O^1^ to O^10^ with increasing size. Established single-particle methods, including negative staining TEM imaging (Figure 2B) and mass photometry (Figure 2C), confirmed the oligomer size estimates from the nanopore experiments. Mass photometry is a recently introduced method based on light scattering that enables oligomer mass analysis at the single-particle level during their interaction with a glass surface.^70,71^Figure 2 demonstrated that the size range of the oligomer population determined by TEM (21–133-mer) and mass photometry (3–103-mer) agrees well with the range established by nanopore experiments (4-mer–122-mer). Nanopore-based analysis revealed two small-sized oligomer populations, called O^1^ and O^2^, corresponding to 4 ± 3-mer and 8 ± 5-mer αSyn oligomers, which were also detected by mass photometry but not by TEM imaging since they were too small (Figure 2). Comparing oligomer size estimations using nanopores to those using TEM and mass photometry shows agreement between the range of estimated sizes among all three single-particle analysis methods and illustrates that the resolution for distinguishing between differently sized oligomers is highest from nanopore experiments, which reveals 10 subpopulations in size, while the resolution of TEM imaging and mass photometry is limited to approximately half the number of subpopulations.

With regard to the relative height of the peaks in the size distribution from the three methods, a direct comparison is difficult due to the difference in resolution between the methods. Comparing similar resolutions by grouping peaks in the histogram from nanopore experiments (Figure 2A) reveals that oligomers with sizes of O^1^ + O^2^ are the most abundant species from nanopore recordings and mass photometry. Since these small-sized oligomers were not detected in TEM, a direct comparison of TEM results with the other two methods is not possible in this size range. Next, oligomers with sizes of O^3^ + O^4^ are the second most abundant ones in both nanopore and mass photometry, and they are the most abundant in TEM (likely because the more abundant, smaller species escape detection). The next most abundant with all three methods is the oligomer size ranges of O^5^ + O^6^ and then of O^7^ + O^8^, and finally O^≥9^. Hence, at comparable size resolution, the three methods agree reasonably well in terms of the relative abundance of differently sized oligomers in the sample.

In addition, we compared the size distribution of αSyn oligomers obtained by nanopore recordings to previously reported sizes of αSyn oligomers. One of the major oligomer populations identified by nanopores labeled as O^2^ consisting of 8 ± 5 monomers agrees well with αSyn oligomers containing 8–16 monomers recently reported to accumulate in the brain of transgenic mouse models with increasing age as determined by a protein complementation approach.^24^ Two other major αSyn oligomer populations labeled as O^3^ and O^4^ consisting of ∼30 and ∼39 monomers agree well with earlier reports of ∼30-mer αSyn oligomers as determined by size exclusion chromatography (SEC) coupled with multiangle laser light scattering (MALLS),^25^ SAXS,^68^ analytical ultracentrifugation (AUC),^72^ and single-molecule photobleaching experiments.^30^ We confirmed the presence of αSyn oligomer populations in this size range by single-particle analysis using TEM and mass photometry and highlighted them with light gray and light green shaded regions in Figure 2B,C. Overall, the peaks in the size distribution from all three single-particle methods indicate that certain sizes of αSyn oligomers are more probable in the sample than others. Among these preferred sizes are oligomers composed of 4 ± 3 monomers (O^1^, Figure 2A,C), 8 ± 5 monomers (O^2^, Figure 2A,C), 25 ± 10 monomers (O^3^, Figure 2A,B,C), 44 ± 4 monomers (O^4^, Figure 2 A,B,C), 57 ± 8 monomers (O^5^, Figure 2A, B, C), 69 ± 8 monomers (O^6^, Figure 2A), 82 ± 5 monomers (O^7^, Figure 2A,B,C), 94 ± 5 monomers (O^8^, Figure 2A), 112 ± 7 monomers (O^9^, Figure 2A), and 122 ± 5 monomers (O^10^, Figure 2A). Hence, starting from tetramers, these preferred sizes appear to be composed of multiples of approximately 12 monomers (i.e., 4, 12, 24, 48, 60, 72, 84, 96, 108, and 120 monomers).

### Analysis of the Shape of Individual αSyn Oligomers

Nanopore-based resistive pulse recording makes it possible to approximate the spheroidal shapes of individual particles by analyzing the current modulations during individual translocation events of single oligomers through the nanopore.^60^ This approximation models particle shape with an ellipsoid with axes *A*, *B*, *B* of the same volume and returns the ratio, *m = A*/*B*, between the axes of an ellipsoid that best represents each individual αSyn oligomer.^66^ To this end, nanopore-based shape analysis exploits the minimum and maximum current blockades (i.e., Δ*I*_min_ and Δ*I*_max_ values, Figure 1B), which correspond to the two extreme orientations of a nonspherical particle with certain volume and shape in the electric field of the nanopore.^60,66^ Earlier studies examining the morphology of αSyn oligomers based on TEM, atomic force microscopy (AFM), and SAXS have revealed oligomers with spherical,^73,74^ annular, toroidal, flattened disc-like, globular, and prolate shapes.^21,25,68,75,76^ Here, based on the Δ*I*_min_ and Δ*I*_max_ values of each translocation event, we model the shape of each oligomer in one attempt as an oblate ellipsoid (i.e., *m* < 1) and in the other attempt as a prolate ellipsoid (i.e., *m* > 1) since solutions for both models can be found for many particles. TEM imaging of the same samples revealed the absence of any large fibrillar or elongated aggregates that may exceed the length of the nanopore (Figure 2B), making it possible to use the same analysis of volume exclusion in the nanopore for characterizing all translocation events.^60,66^ In addition, we confirmed that nanopore recording at the high ionic strength (i.e., 2 M KCl) that we used does not change the size distribution of αSyn oligomers during the experiment compared to solutions with physiologic ionic strength (Supplementary Note 2 and Supporting Information Figures S2 and S3). Nonetheless, different buffer conditions (pH, ionic strength, type and valency of salt), as well as preparation methods and conditions, can strongly affect the size and shape distribution of αSyn oligomers; hence the comparison of the size and shape values determined in this work with those from previous studies primarily illustrates that the results here are in the range of previous reports and that the method reported here is capable of characterizing this commonly observed range. Agreement between the sizes and shapes reported here with those reported from other preparation methods does not imply that these particles necessarily are the same in other aspects as well. All comparisons of size and shape should be viewed with these considerations in mind.

**Figure 3:**
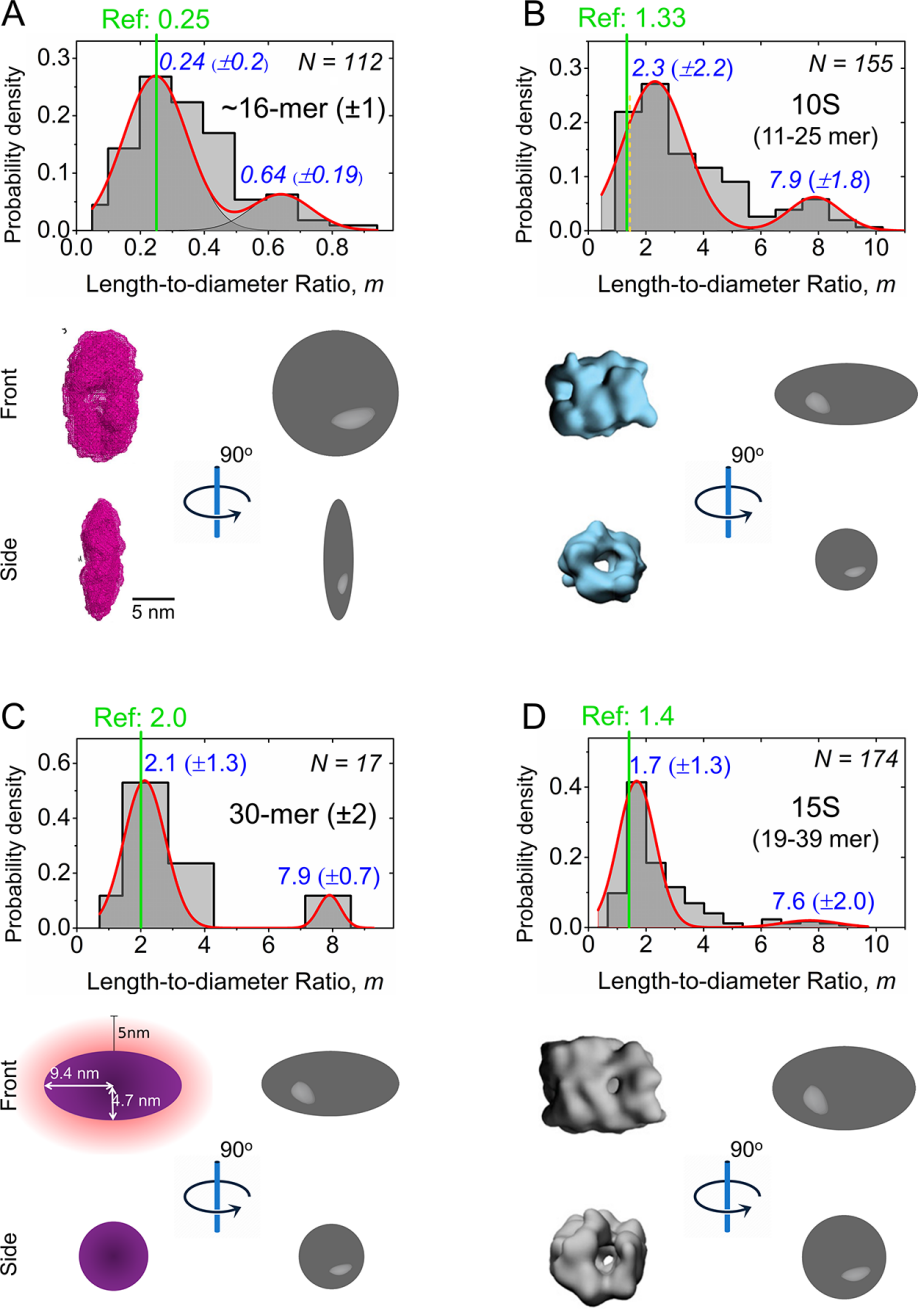
Nanopore-based shape approximation of αSyn oligomers with sizes in a range that was previously reported to contain toxic oligomers. (A) Distribution of the length-to-diameter ratio, *m*, of oligomers consisting of 15–17 αSyn monomers, as obtained from nanopore-based shape analysis of resistive pulses from single αSyn oligomers in solution. The cartoon below shows a comparison of the approximated ellipsoidal shape of a ∼16-mer determined by nanopore (gray ellipsoid, top, and side view) with the structure of the 16-mer revealed by SAXS experiments (mesh representation, in pink). Adapted with permission from ref (68). Copyright 2011, National Academy of Sciences. (B) Distribution of the length-to-diameter ratio of 10S oligomers consisting of 11–25 αSyn monomers as obtained from a nanopore-based shape analysis. The cartoon below shows a comparison of the approximated ellipsoidal shape as determined by nanopore (gray spheroids) with the three-dimensional structures for 10S oligomers determined by cryo-EM (blue). Reprinted with permission from ref (65). Copyright 2015, National Academy of Sciences. The dashed line in yellow marks the first bin of the histogram, which represents the second most probable bin of the shape distribution for this oligomer size range (11–25 monomers) as determined by nanopore experiments and agrees well with the previously reported shape value (green line).^65^ (C) Distribution of the length-to-diameter ratio of 30 ± 2-mer oligomers as obtained from nanopore-based shape analysis. The cartoon below shows a comparison of the approximate ellipsoidal shape of 30-mers as determined by nanopore (gray spheroids) with an ellipsoidal model of the 30-mer determined by SAXS (purple). Adapted with permission from ref (25). Copyright 2014, American Chemical Society. (D) Distribution of the length-to-diameter ratio of 15S^65^ oligomers consisting of 19–39 αSyn monomers as obtained from nanopore-based shape analysis. Comparison of the approximate ellipsoidal shape as determined by nanopore (gray spheroids) with the three-dimensional structures for 15S oligomers determined by cryo-EM (left). Reprinted with permission from ref (65). Copyright 2015, National Academy of Sciences. The most probable *m* value of each subpopulation is shown at the top in blue. The solid lines in light green show the reference length-to-diameter ratio, *m*, for the respective oligomeric species.

Nanopore-based simultaneous determination of oligomer size and shape of each particle provides the opportunity to characterize oligomer subpopulations with respect to these two important parameters. For instance, we determined the shape of αSyn oligomers in a size range that was previously described to include toxic oligomers (see Figure 3A–D).^21,24,68^ Giehm et al. used SAXS experiments to determine a “wreath shape” with an average length-to-diameter ratio, *m*, of 0.25 of so-called “vesicle disrupting α-synuclein oligomers” consisting of ∼16 monomers.^68^Figure 3A shows the distribution of *m* values obtained for the oblate model of 16 ± 1-mer oligomers by nanopore experiments and reveals that the major oligomer subpopulation corresponds to an oblate shape value of *m* = 0.24, in excellent agreement with the reported shape of 0.25 from SAXS experiments.^68^ In addition to this dominant subpopulation, nanopore recordings identified a second, less prominent subpopulation with a shape value of *m* = 0.64 in the same sample. Due to its low abundance, this subpopulation may not be detectable in SAXS-based ensemble analysis.

Figure 3B and D show the distribution of the approximated shape of oligomers with sizes that match those of so-called “kinetically trapped toxic α-synuclein oligomers”, which include 10S (11–25-mer) and 15S (19–39-mer) aggregates. These have previously been characterized as prolates with average values of *m* = 2.0 for 10S oligomers and *m* = 1.4 for 15S oligomers based on their sedimentation coefficients.^65^ The nanopore experiments reveal a dominant oligomer subpopulation with a length-to-diameter ratio, *m*, of 2.3 for oligomers in the 10S size range and a dominant oligomer subpopulation with a length-to-diameter ratio, *m*, of 1.7 for oligomers in the 15S size range, in good agreement with the previously reported average shape values.^65^

In addition to these studies, Lorenzen et al. used size exclusion chromatography in combination with multiangle scattering and SAXS and determined an average prolate ellipsoid shape with an *m* value of 2.0 for oligomers consisting of 30 ± 2 monomers.^25^ The nanopore experiments carried out here indicate a major subpopulation with an *m* value of 2.1 in the distribution of the length-to-diameter ratio of similar-sized 30-mer oligomers (Figure 3C), again in excellent agreement with the reported average shape value. Overall, Figure 3 indicates that for oligomeric species within size brackets that have previously been reported as toxic, nanopore recordings resolve typically one dominant subpopulation in oligomer shape that agrees well with the previous reports as well as a less prominent subpopulation of oligomer shape that might be missed using ensemble analysis. As an alternative possibility, the additional, less prominent subpopulation of oligomer shapes observed in all four size ranges could be the effect of different experimental conditions used in the work presented here compared to the other methods.

### Quantifying the Abundance of αSyn Oligomers

**Figure 4:**
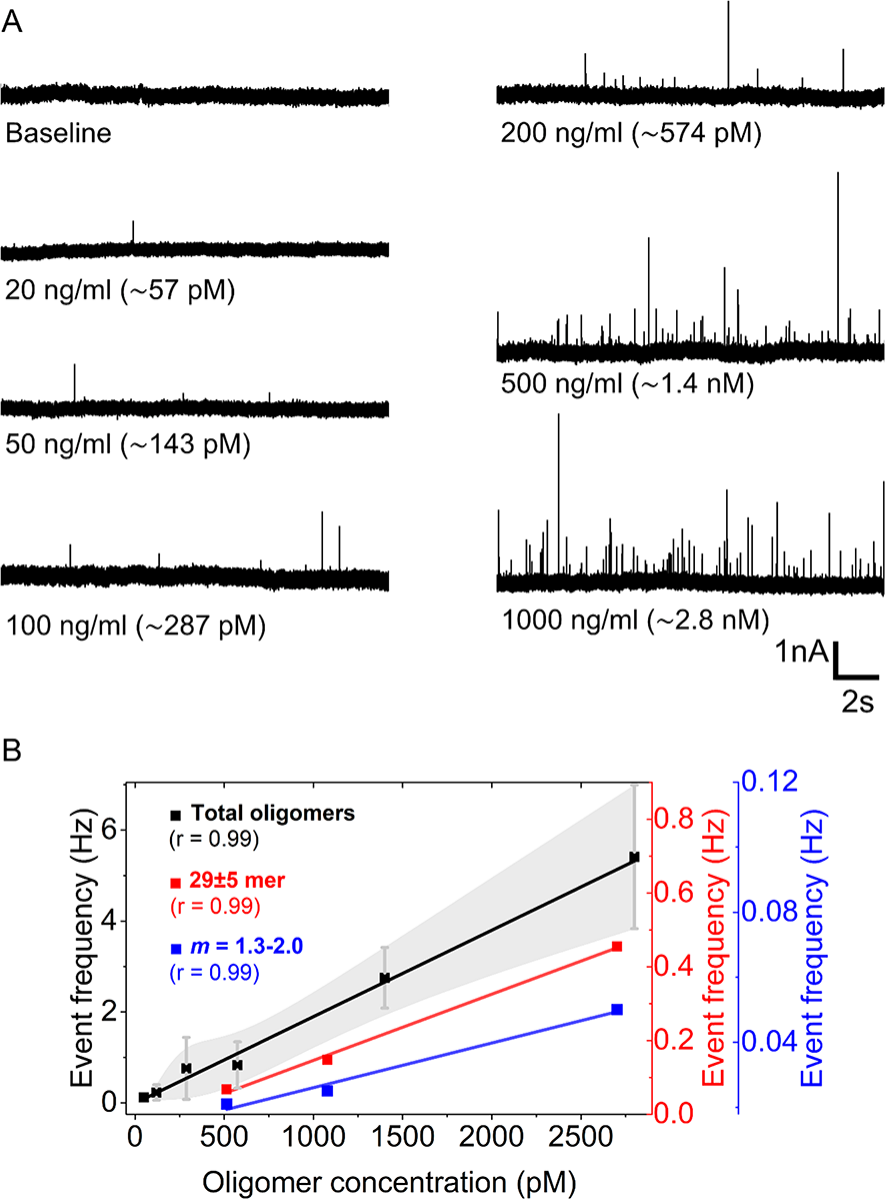
Quantification of the abundance of αSyn oligomers using nanopores. (A) Original current recordings (∼20 s) low-pass filtered with a cutoff frequency of 50 kHz showing resistive pulses (upward spikes) due to translocation of individual αSyn oligomers at increasing total concentrations ranging from 50 pM to 2.4 nM. (B) The frequency of translocation events at different total oligomer concentrations (black) and frequency of oligomer subpopulations with specific size (29 ± 5-monomers, red) or specific shape (length-to-diameter ratio, *m*, ranging from 1.3 to 2.0, blue). Translocation event frequency is plotted as squares (black, red, and blue), and the red solid line is a linear fit (slope of 0.002 Hz/pM and Pearson’s *r* of 0.99). The shaded region (light gray) shows the standard deviation of four different measurements using a different nanopore chip for each measurement.

In order to assess if the nanopore-based analysis is able to quantify the total concentration of αSyn oligomers as well as the concentration of subpopulations, we monitored the frequency of individual translocation events through nanopores at increasing total oligomer concentrations ranging from picomolar to nanomolar. Based on previous work, translocation frequency is proportional to the concentration of detectable particles.^77^Figure 4A and B show that, as expected,^77^ the translocation event frequency is directly proportional to the total concentration of αSyn oligomers at least up to ∼2.5 nM (see Figure 4B). This range of sensitivity is beneficial for assessment of the concentration of αSyn oligomers as a potential biomarker for PD. We propose that PD biomarker analysis will benefit from the ability to count individual oligomer translocations using the nanopore-based method because it is not limited to quantifying the total concentration of oligomers but also makes it possible to identify and quantify the fraction of subpopulations. For instance, it can determine the concentration of αSyn oligomers of certain sizes such as 8–10-mers, 21–39-mers, and 40–50-mers, which are considered to include toxic oligomers. Or it can quantify the fraction of oligomers with a certain shape or the combination of a certain size and shape. Figure 4B shows, for instance, that the frequency of translocations from oligomers consisting of 29 ± 5-monomers or from oligomers with a shape value ranging from 1.4 to 2.0 (a range that includes shape values of tubular oligomers such as *m* = 1.3, 1.4, and 2.0 that may contain toxic oligomers^25,65^) also increased linearly with total oligomer concentration. This capability of quantifying specific subpopulations is a direct benefit of the characterization of each individual translocation event with respect to the size and shape of the oligomer particle that caused the resistive pulse combined with its measured frequency of occurrence.

Nanopore-based analysis, therefore, provides a proof of principle for characterization and quantification of amyloid oligomers at the single-oligomer level in a mixture that is heterogeneous with respect to oligomer size and shape. Measuring unmodified oligomers in solution ensures minimal perturbation of the inherent biophysical properties of these aggregates with the possibility of simultaneous oligomer size and shape analysis of each particle. In addition, the recent development of hydrogel-interfaced nanopores combined with asymmetric salt concentrations may enable quantification of particle concentrations in the femtomolar range^69^ and in physiological buffers,^78^ therefore establishing characteristics that enable biomarker analysis.

## Conclusions

The work presented here demonstrates that nanopore-based characterization of αSyn oligomers can overcome at least four of the current challenges in oligomer characterization using conventional methods by providing: (i) analysis of the size and shape of oligomers simultaneously, (ii) characterization of oligomers on a single-particle level, thereby circumventing the complications from averaging heterogeneous samples, (iii) measurements in solution (albeit at high ionic strength) and in a label-free manner, thereby examining oligomers in their hydrated, unmodified state, and (iv) quantification of concentrations of total oligomer content or of specific subpopulations in size and or shape that may contain particularly toxic or diagnostically relevant species. Since oligomers presumably represent the key neurotoxic species,^8,9,21^ a quantitative understanding of their abundance, physical properties (i.e., size and shape), and heterogeneity in solution is essential to elucidate their structure–toxicity relationship. The nanopore-based approach presented here enables size and shape estimation of oligomers at the single-particle level, in real time with minimal sample perturbation, and it characterizes oligomer samples within tens of minutes based on hundreds of translocation events; even at a total oligomer concentration as low as 150 pM, more than 100 translocation events are recorded within 10 min.

Determination of size distributions by the nanopore-based approach revealed 10 stable subpopulations of oligomers that contain multiples of approximately 12 monomers as well as approximations of their shape on a single-particle level. The achieved size resolution is twice that of mass photometry and TEM analysis (see Supplementary Tables S1 and S2). In addition, TEM-based characterization of oligomers and its potential use for biomarker analysis is typically not available in clinical settings, is expensive, and may involve artifact-prone dry-state sample preparation. Importantly, these established single-particle methods do not have the capability of determining the shape of individual oligomeric species. Although the nanopore-based approach presented here currently requires specialized training and expertise, it provides a rapid, quantitative, label-free, and cost-effective multiparametric characterization of heterogeneous amyloid oligomers in solution. These benefits address the urgent need for methods that make it possible to quantify heterogeneous amyloid oligomers as biomarkers or for testing drug to treat neurodegenerative disorders.^35,39,79^

## Methods/Experimental

### Materials

Oligomers of α-synuclein were obtained from ND BioSciences, Switzerland, as a part of a collaboration supported by the Michael J. Fox Foundation (MJFF-009813). The preparation of these αSyn oligomers has been discussed in detail by Kumar et al.^67^ Oligomers of αSyn from ND Biosciences were aliquoted, flash frozen in liquid N_2_, stored at −80 °C upon arrival, and taken out only before the measurements. We obtained the EM-Tec Formvar Carbon support film on copper 200 square mesh (cat. no. 22-1MFC20-100) for TEM imaging of αSyn oligomers from the company Micro to Nano, Netherlands. All the nanopores used in this work were purchased (prefabricated by helium ion milling) from Norcada Inc., Canada, or fabricated by helium ion beam sculpting, as explained previously at the University of Arkansas, USA.^80^ The prefabricated nanopore chips (4 × 4 mm) from Norcada contained a 10 × 10 μm SiN_*x*_ window in a 30 nm thick free-standing SiN_*x*_ membrane. The chips contained a 100 nm thick SiO_2_ underlayer to reduce capacitive noise. We purchased all other chemicals and buffers from Sigma-Aldrich unless otherwise stated.

### Resistive Pulse Sensing Using Nanopores

We carried out all resistive pulse sensing experiments using a recording buffer containing 2 M KCl and 10 mM HEPES buffer (pH 7.4) as described earlier by Yusko et al.^60^ and Houghtaling et al.^66^ We used polymer-coated solid-state nanopores with a diameter of 25, 30, or 56 nm to characterize αSyn oligomers in free translocation mode as described earlier.^66^ As described in detail previously, the antifouling polymer surface coating with PAcrAm-*g*-PMOXA polymer (SuSoS AG, Switzerland) minimized nonspecific interactions between analytes and the pore wall, facilitating free translocation through nanopores with minimal interactions with the pore wall and hence minimal perturbations.^57^ We applied a constant potential of ±100 mV across the nanopore and then measured the current at a sampling rate of 500 kHz via a USB-6361 data acquisition card using an AxoPatch 200B patch-clamp amplifier (Molecular Devices) in voltage-clamp mode (β = 1) in combination with LabVIEW (National Instruments) software. We filtered the acquired data with a Gaussian low-pass filter at a cutoff frequency of 50 kHz. We performed a threshold search (5× the standard deviation of the baseline current) for resistive pulses within the current recordings and determined the oligomer size (as volume) and shape (as length-to-diameter ratio, *m*) by so-called “intra-event analysis” as described earlier.^60,66^ For the analysis of shape with the previously described convolution algorithm,^60,66^ we constrained the spread “σ” of the fitting procedure to be greater than, or equal to, the standard deviation of baseline noise. We used ferritin, an almost perfectly spherical protein, as a standard to identify the best nanopores, which accurately estimated the size and spherical shape of ferritin and could then be used to determine the size and shape of Syn oligomers (see Figure 1C). During the size and shape analysis of Syn oligomers, we only used nanopores that gave a spherical shape value for ferritin (i.e., with an *m* value close to 1).

### Transmission Electron Microscopy

Carbon-coated 300-mesh copper grids (Electron Microscopy Sciences, Hatfield) were plasma cleaned for 5 s using an oxygen plasma cleaner (Zepto RIE, Dienner), before pipetting 5 μL of Syn oligomers sample (∼100 μg/mL concentration) in PBS buffer, pH 7.4, on top of the grids followed by 2 min of incubation. The grids were washed with a water droplet. Uranyl acetate (2% w/v) was added (3 μL), and the mixture was incubated for 2 min. Excess stain was blotted off with filter paper and dried. TEM images were recorded with an FEI Tecnai Spirit operating at 120 kV. We used ImageJ for particle size analysis of Syn oligomers.^81^

### Mass Photometry

We acquired all the mass photometry data using a TwoMP mass photometer (Refeyn Ltd., Oxford, UK) similar to that described previously by Sonn-Segev et al. with slight modifications.^70^ Briefly, microscope coverslips (24 × 50 mm Thorlabs, cat. no. CG15KH1) cleaned using isopropanol followed by pure water thrice sequentially were used along with PDMS CultureWell gaskets (cat. no. GBL103250) for single-oligomer mass analysis. We diluted αSyn oligomers up to a concentration of ∼250 ng/mL in PBS or in 2 M KCl immediately prior to mass photometry measurements. To determine the effect of the ionic strength, αSyn oligomers were incubated for 90 min or overnight in PBS or 2 M KCl at room temperature. For each mass photometry acquisition, 20 μL of diluted protein was introduced into the chamber, and following autofocus stabilization, movies of 180 s duration were recorded. Each sample was measured at least three times independently (*n* ≥ 3). Mass photometry data acquisition was performed using AcquireMP (Refeyn Ltd., v2.4.1). All mass photometry images were processed and analyzed using DiscoverMP (Refeyn Ltd., v2.5.0).

## Supporting Information Available

Details of αSyn monomer volume analysis (Note 1), details of αSyn oligomer stability during nanopore recordings (Note 2), oligomer size and shape estimation (Note 3), results of αSyn monomer volume estimates by resistive pulse sensing (Figure S1), results of αSyn oligomer size analysis after incubation in PBS and recording buffer determined by TEM (Figure S2) and mass photometry (Figure S3), αSyn oligomer size distributions determined using different size nanopores (Figure S4), oligomer size analysis using three different methods (Table S1), comparison of the different methods used in this study (Table S2) (PDF)

## Notes

Preprint available at: Saurabh Awasthi, Cuifeng Ying, Jiali Li, Michael Mayer, Simultaneous Determination of the Size and Shape of Single α-Synuclein Oligomers in Solution, 2023, 2023.01.09.523202, BioRxiv, https://doi.org/10.1101/2023.01.09.523202 (accessed May 26, 2023).

## References

[ref1] Galvin J. E.; Lee V. M.-Y.; Trojanowski J. Q. Synucleinopathies: Clinical and pathological implications. Arch Neurol. 2001, 58 (2), 186–190. 10.1001/archneur.58.2.186. 11176955

[ref2] Goedert M.; Jakes R.; Spillantini M. G. The Synucleinopathies: Twenty years on. J. Parkinson’s Dis. 2017, 7, S51–S69. 10.3233/JPD-179005. 28282814 PMC5345650

[ref3] McCann H.; Stevens C. H.; Cartwright H.; Halliday G. M. α-Synucleinopathy phenotypes. Parkinsonism Relat. Disord. 2014, 20, S62–S67. 10.1016/S1353-8020(13)70017-8. 24262191

[ref4] Stefanis L. α-Synuclein in Parkinson’s disease. Cold Spring Harbor Perspect. Med. 2012, 2 (2), a009399–a009399. 10.1101/cshperspect.a009399. PMC3281589 22355802

[ref5] Kalia L. V.; Kalia S. K.; McLean P. J.; Lozano A. M.; Lang A. E. Alpha-Synuclein oligomers and clinical implications for Parkinson disease. Ann. Neurol. 2013, 73 (2), 155–69. 10.1002/ana.23746. 23225525 PMC3608838

[ref6] Winner B.; Jappelli R.; Maji S. K.; Desplats P. A.; Boyer L.; Aigner S.; Hetzer C.; Loher T.; Vilar M.; Campioni S.; Tzitzilonis C.; Soragni A.; Jessberger S.; Mira H.; Consiglio A.; Pham E.; Masliah E.; Gage F. H.; Riek R. In vivo demonstration that alpha-synuclein oligomers are toxic. Proc. Natl. Acad. Sci. U. S. A. 2011, 108 (10), 4194–9. 10.1073/pnas.1100976108. 21325059 PMC3053976

[ref7] Martin Z. S.; Neugebauer V.; Dineley K. T.; Kayed R.; Zhang W.; Reese L. C.; Taglialatela G. α-Synuclein oligomers oppose long-term potentiation and impair memory through a calcineurin-dependent mechanism: relevance to human synucleopathic diseases. J. Neurochem. 2012, 120 (3), 440–452. 10.1111/j.1471-4159.2011.07576.x. 22060133 PMC3253169

[ref8] van Diggelen F.; Hrle D.; Apetri M.; Christiansen G.; Rammes G.; Tepper A.; Otzen D. E. Two conformationally distinct α-synuclein oligomers share common epitopes and the ability to impair long-term potentiation. PLoS One 2019, 14 (3), e0213663 10.1371/journal.pone.0213663. 30901378 PMC6430514

[ref9] Emin D.; Zhang Y. P.; Lobanova E.; Miller A.; Li X.; Xia Z.; Dakin H.; Sideris D. I.; Lam J. Y. L.; Ranasinghe R. T.; Kouli A.; Zhao Y.; De S.; Knowles T. P. J.; Vendruscolo M.; Ruggeri F. S.; Aigbirhio F. I.; Williams-Gray C. H.; Klenerman D. Small soluble α-synuclein aggregates are the toxic species in Parkinson’s disease. Nat. Commun. 2022, 13 (1), 5512 10.1038/s41467-022-33252-6. 36127374 PMC9489799

[ref10] Colla E.; Jensen P. H.; Pletnikova O.; Troncoso J. C.; Glabe C.; Lee M. K. Accumulation of toxic α-synuclein oligomer within endoplasmic reticulum occurs in α-synucleinopathy In Vivo. J. Neurosci. 2012, 32 (10), 3301–3305. 10.1523/JNEUROSCI.5368-11.2012. 22399752 PMC3548448

[ref11] Prots I.; Grosch J.; Brazdis R.-M.; Simmnacher K.; Veber V.; Havlicek S.; Hannappel C.; Krach F.; Krumbiegel M.; Schütz O.; Reis A.; Wrasidlo W.; Galasko D. R.; Groemer T. W.; Masliah E.; Schlötzer-Schrehardt U.; Xiang W.; Winkler J.; Winner B. α-Synuclein oligomers induce early axonal dysfunction in human iPSC-based models of synucleinopathies. Proc. Natl. Acad. Sci. U. S. A. 2018, 115 (30), 7813–7818. 10.1073/pnas.1713129115. 29991596 PMC6065020

[ref12] Breydo L.; Wu J. W.; Uversky V. N. α-Synuclein misfolding and Parkinson’s disease. Biochim. Biophys. Acta, Mol. Basis Dis. 2012, 1822 (2), 261–285. 10.1016/j.bbadis.2011.10.002. 22024360

[ref13] Tokuda T.; Qureshi M. M.; Ardah M. T.; Varghese S.; Shehab S. A.; Kasai T.; Ishigami N.; Tamaoka A.; Nakagawa M.; El-Agnaf O. M. Detection of elevated levels of alpha-synuclein oligomers in CSF from patients with Parkinson disease. Neurology 2010, 75 (20), 1766–72. 10.1212/WNL.0b013e3181fd613b. 20962290

[ref14] Hansson O.; Hall S.; Ohrfelt A.; Zetterberg H.; Blennow K.; Minthon L.; Nagga K.; Londos E.; Varghese S.; Majbour N. K.; Al-Hayani A.; El-Agnaf O. M. Levels of cerebrospinal fluid alpha-synuclein oligomers are increased in Parkinson’s disease with dementia and dementia with Lewy bodies compared to Alzheimer’s disease. Alzheimer’s Res. Ther. 2014, 6 (3), 25 10.1186/alzrt255. 24987465 PMC4075410

[ref15] van Steenoven I.; Majbour N. K.; Vaikath N. N.; Berendse H. W.; van der Flier W. M.; van de Berg W. D. J.; Teunissen C. E.; Lemstra A. W.; El-Agnaf O. M. A. α-Synuclein species as potential cerebrospinal fluid biomarkers for dementia with lewy bodies. Mov. Disord. 2018, 33 (11), 1724–1733. 10.1002/mds.111. 30440090 PMC6519232

[ref16] Ugalde C. L.; Lawson V. A.; Finkelstein D. I.; Hill A. F. The role of lipids in α-synuclein misfolding and neurotoxicity. J. Biol. Chem. 2019, 294 (23), 9016–9028. 10.1074/jbc.REV119.007500. 31064841 PMC6556586

[ref17] Iyer A.; Claessens M. M. A. E. Disruptive membrane interactions of alpha-synuclein aggregates. Biochim. Biophys. Acta, Proteins Proteomics 2019, 1867 (5), 468–482. 10.1016/j.bbapap.2018.10.006. 30315896

[ref18] Fusco G.; Chen S. W.; Williamson P. T. F.; Cascella R.; Perni M.; Jarvis J. A.; Cecchi C.; Vendruscolo M.; Chiti F.; Cremades N.; Ying L.; Dobson C. M.; De Simone A. Structural basis of membrane disruption and cellular toxicity by α-synuclein oligomers. Science 2017, 358 (6369), 1440–1443. 10.1126/science.aan6160. 29242346

[ref19] Celej M. S.; Sarroukh R.; Goormaghtigh E.; Fidelio; Gerardo D.; Ruysschaert J.-M.; Raussens V. Toxic prefibrillar α-synuclein amyloid oligomers adopt a distinctive antiparallel β-sheet structure. Biochem. J. 2012, 443 (3), 719–726. 10.1042/BJ20111924. 22316405

[ref20] González-Lizárraga F.; Socías S. B.; Ávila C. L.; Torres-Bugeau C. M.; Barbosa L. R. S.; Binolfi A.; Sepúlveda-Díaz J. E.; Del-Bel E.; Fernandez C. O.; Papy-Garcia D.; Itri R.; Raisman-Vozari R.; Chehín R. N. Repurposing doxycycline for synucleinopathies: remodelling of α-synuclein oligomers towards non-toxic parallel beta-sheet structured species. Sci. Rep. 2017, 7 (1), 41755 10.1038/srep41755. 28155912 PMC5290535

[ref21] Chen S. W.; Drakulic S.; Deas E.; Ouberai M.; Aprile F. A.; Arranz R.; Ness S.; Roodveldt C.; Guilliams T.; De-Genst E. J.; Klenerman D.; Wood N. W.; Knowles T. P. J.; Alfonso C.; Rivas G.; Abramov A. Y.; Valpuesta J. M.; Dobson C. M.; Cremades N. Structural characterization of toxic oligomers that are kinetically trapped during α-synuclein fibril formation. Proc. Natl. Acad. Sci. U. S. A. 2015, 112 (16), E1994–E2003. 10.1073/pnas.1421204112. 25855634 PMC4413268

[ref22] Cremades N.; Cohen S. I. A.; Deas E.; Abramov A. Y.; Chen A. Y.; Orte A.; Sandal M.; Clarke R. W.; Dunne P.; Aprile F. A.; Bertoncini C. W.; Wood N. W.; Knowles T. P. J.; Dobson C. M.; Klenerman D. Direct observation of the interconversion of normal and toxic forms of α-synuclein. Cell 2012, 149 (5), 1048–1059. 10.1016/j.cell.2012.03.037. 22632969 PMC3383996

[ref23] Ries H. M.; Nussbaum-Krammer C. Shape matters: the complex relationship between aggregation and toxicity in protein-misfolding diseases. Essays Biochem. 2016, 60 (2), 181–190. 10.1042/EBC20160008. 27744334

[ref24] Kiechle M.; von Einem B.; Höfs L.; Voehringer P.; Grozdanov V.; Markx D.; Parlato R.; Wiesner D.; Mayer B.; Sakk O.; Baumann B.; Lukassen S.; Liss B.; Ekici A. B.; Ludolph A. C.; Walther P.; Ferger B.; McLean P. J.; Falkenburger B. H.; Weishaupt J. H.; Danzer K. M. In Vivo Protein complementation demonstrates presynaptic α-synuclein oligomerization and age-dependent accumulation of 8–16-mer oligomer species. Cell Rep. 2019, 29 (9), 2862–2874.e9. 10.1016/j.celrep.2019.10.089. 31775051

[ref25] Lorenzen N.; Nielsen S. B.; Buell A. K.; Kaspersen J. D.; Arosio P.; Vad B. S.; Paslawski W.; Christiansen G.; Valnickova-Hansen Z.; Andreasen M.; Enghild J. J.; Pedersen J. S.; Dobson C. M.; Knowles T. P. J.; Otzen D. E. The role of stable α-synuclein oligomers in the molecular events underlying amyloid formation. J. Am. Chem. Soc. 2014, 136 (10), 3859–3868. 10.1021/ja411577t. 24527756

[ref26] Danzer K. M.; Ruf W. P.; Putcha P.; Joyner D.; Hashimoto T.; Glabe C.; Hyman B. T.; McLean P. J. Heat-shock protein 70 modulates toxic extracellular α-synuclein oligomers and rescues trans-synaptic toxicity. FASEB J. 2011, 25 (1), 326–336. 10.1096/fj.10-164624. 20876215 PMC3005424

[ref27] Wägele J.; De Sio S.; Voigt B.; Balbach J.; Ott M. How fluorescent tags modify oligomer size distributions of the Alzheimer peptide. Biophys. J. 2019, 116 (2), 227–238. 10.1016/j.bpj.2018.12.010. 30638607 PMC6350010

[ref28] Zhang S.; Fox D. M.; Urbanc B. Elucidating the role of hydroxylated phenylalanine in the formation and structure of cross-linked Aβ oligomers. J. Phys. Chem. B 2019, 123 (5), 1068–1084. 10.1021/acs.jpcb.8b12120. 30642171

[ref29] Arter W. E.; Xu C. K.; Castellana-Cruz M.; Herling T. W.; Krainer G.; Saar K. L.; Kumita J. R.; Dobson C. M.; Knowles T. P. J. Rapid structural, kinetic, and immunochemical analysis of alpha-synuclein oligomers in solution. Nano Lett. 2020, 20 (11), 8163–8169. 10.1021/acs.nanolett.0c03260. 33079553 PMC7116857

[ref30] Zijlstra N.; Blum C.; Segers-Nolten I. M. J.; Claessens M. M. A. E.; Subramaniam V. Molecular composition of sub-stoichiometrically labeled α-synuclein oligomers determined by single-molecule photobleaching. Angew. Chem., Int. Ed. Engl. 2012, 51 (35), 8821–8824. 10.1002/anie.201200813. 22806998

[ref31] Sierecki E.; Giles N.; Bowden Q.; Polinkovsky M. E.; Steinbeck J.; Arrioti N.; Rahman D.; Bhumkar A.; Nicovich P. R.; Ross I.; Parton R. G.; Böcking T.; Gambin Y. Nanomolar oligomerization and selective co-aggregation of α-synuclein pathogenic mutants revealed by single-molecule fluorescence. Sci. Rep. 2016, 6 (1), 37630 10.1038/srep37630. 27892477 PMC5385372

[ref32] Lau D.; Magnan C.; Hill K.; Cooper A.; Gambin Y.; Sierecki E. Single Molecule fingerprinting reveals different amplification properties of α-synuclein oligomers and preformed fibrils in seeding assay. ACS Chem. Neuro. 2022, 13 (7), 883–896. 10.1021/acschemneuro.1c00553. PMC8990999 35286811

[ref33] El-Agnaf O. M.; Salem S. A.; Paleologou K. E.; Curran M. D.; Gibson M. J.; Court J. A.; Schlossmacher M. G.; Allsop D. Detection of oligomeric forms of alpha-synuclein protein in human plasma as a potential biomarker for Parkinson’s disease. FASEB J. 2006, 20 (3), 419–25. 10.1096/fj.03-1449com. 16507759

[ref34] Hansson O.; Hall S.; Öhrfelt A.; Zetterberg H.; Blennow K.; Minthon L.; Nägga K.; Londos E.; Varghese S.; Majbour N. K.; Al-Hayani A.; El-Agnaf O. M. A. Levels of cerebrospinal fluid α-synuclein oligomers are increased in Parkinson’s disease with dementia and dementia with Lewy bodies compared to Alzheimer’s disease. Alzheimer’s Res. Ther. 2014, 6 (3), 25 10.1186/alzrt255. 24987465 PMC4075410

[ref35] Majbour N. K.; Vaikath N. N.; van Dijk K. D.; Ardah M. T.; Varghese S.; Vesterager L. B.; Montezinho L. P.; Poole S.; Safieh-Garabedian B.; Tokuda T.; Teunissen C. E.; Berendse H. W.; van de Berg W. D. J.; El-Agnaf O. M. A. Oligomeric and phosphorylated alpha-synuclein as potential CSF biomarkers for Parkinson’s disease. Mol. Neurodeg. 2016, 11 (1), 7 10.1186/s13024-016-0072-9. PMC4717559 26782965

[ref36] Lassen L. B.; Gregersen E.; Isager A. K.; Betzer C.; Kofoed R. H.; Jensen P. H. ELISA method to detect α-synuclein oligomers in cell and animal models. PLoS One 2018, 13 (4), e0196056 10.1371/journal.pone.0196056. 29698510 PMC5919555

[ref37] Tokuda T.; Qureshi M. M.; Ardah M. T.; Varghese S.; Shehab S. A. S.; Kasai T.; Ishigami N.; Tamaoka A.; Nakagawa M.; El-Agnaf O. M. A. Detection of elevated levels of α-synuclein oligomers in CSF from patients with Parkinson disease. Neurology. 2010, 75 (20), 1766–1770. 10.1212/WNL.0b013e3181fd613b. 20962290

[ref38] Kumar S. T.; Jagannath S.; Francois C.; Vanderstichele H.; Stoops E.; Lashuel H. A. How specific are the conformation-specific α-synuclein antibodies? Characterization and validation of 16 α-synuclein conformation-specific antibodies using well-characterized preparations of α-synuclein monomers, fibrils and oligomers with distinct structures and morphology. Neurobiol. dis. 2020, 146, 105086 10.1016/j.nbd.2020.105086. 32971232

[ref39] Kulenkampff K.; Wolf Perez A.-M.; Sormanni P.; Habchi J.; Vendruscolo M. Quantifying misfolded protein oligomers as drug targets and biomarkers in Alzheimer and Parkinson diseases. Nat. Rev. Chem. 2021, 5 (4), 277–294. 10.1038/s41570-021-00254-9. 37117282

[ref40] Blömeke L.; Pils M.; Kraemer-Schulien V.; Dybala A.; Schaffrath A.; Kulawik A.; Rehn F.; Cousin A.; Nischwitz V.; Willbold J.; Zack R.; Tropea T. F.; Bujnicki T.; Tamgüney G.; Weintraub D.; Irwin D.; Grossman M.; Wolk D. A.; Trojanowski J. Q.; Bannach O.; Chen-Plotkin A.; Willbold D. Quantitative detection of α-Synuclein and Tau oligomers and other aggregates by digital single particle counting. npj Parkinson’s Dis. 2022, 8 (1), 68 10.1038/s41531-022-00330-x. 35655068 PMC9163356

[ref41] Wang H. Y.; Gu Z.; Cao C.; Wang J.; Long Y. T. Analysis of a single alpha-synuclein fibrillation by the interaction with a protein nanopore. Anal. Chem. 2013, 85 (17), 8254–61. 10.1021/ac401496x. 23899046

[ref42] Li X.; Tong X.; Lu W.; Yu D.; Diao J.; Zhao Q. Label-free detection of early oligomerization of α-synuclein and its mutants A30P/E46K through solid-state nanopores. Nanoscale 2019, 11 (13), 6480–6488. 10.1039/C9NR00023B. 30892349

[ref43] Giamblanco N.; Fichou Y.; Janot J.-M.; Balanzat E.; Han S.; Balme S. Mechanisms of heparin-induced tau aggregation revealed by a single nanopore. ACS Sens. 2020, 5 (4), 1158–1167. 10.1021/acssensors.0c00193. 32216272

[ref44] Yu R.-J.; Lu S.-M.; Xu S.-W.; Li Y.-J.; Xu Q.; Ying Y.-L.; Long Y.-T. Single molecule sensing of amyloid-β aggregation by confined glass nanopores. Chem. Sci. 2019, 10 (46), 10728–10732. 10.1039/C9SC03260F. 32153747 PMC7020925

[ref45] Hu R.; Diao J.; Li J.; Tang Z.; Li X.; Leitz J.; Long J.; Liu J.; Yu D.; Zhao Q. Intrinsic and membrane-facilitated α-synuclein oligomerization revealed by label-free detection through solid-state nanopores. Sci. Rep. 2016, 6, 20776 10.1038/srep20776. 26865505 PMC4749980

[ref46] Abrao-Nemeir I.; Bentin J.; Meyer N.; Janot J.-M.; Torrent J.; Picaud F.; Balme S. Investigation of α-synuclein and amyloid-β(42)-E22Δ oligomers using SiN nanopore functionalized with L-Dopa. Chem. - Asian J. 2022, 17 (20), e202200726 10.1002/asia.202200726. 36038502 PMC9826174

[ref47] Meyer N.; Janot J.-M.; Torrent J.; Balme S. Real-time fast amyloid seeding and translocation of α-synuclein with a nanopipette. ACS Cent. Sci. 2022, 8 (4), 441–448. 10.1021/acscentsci.1c01404. 35505874 PMC9052795

[ref48] Yusko E. C.; Johnson J. M.; Majd S.; Prangkio P.; Rollings R. C.; Li J.; Yang J.; Mayer M. Controlling protein translocation through nanopores with bio-inspired fluid walls. Nat. Nanotechnol. 2011, 6, 253 10.1038/nnano.2011.12. 21336266 PMC3071889

[ref49] Houghtaling J.; List J.; Mayer M. Nanopore-Based, Rapid characterization of individual amyloid particles in solution: Concepts, challenges, and prospects. Small 2018, 14 (46), 1802412 10.1002/smll.201802412. 30225962

[ref50] Tavassoly O.; Kakish J.; Nokhrin S.; Dmitriev O.; Lee J. S. The use of nanopore analysis for discovering drugs which bind to alpha-synuclein for treatment of Parkinson’s disease. Eur. J. Med. Chem. 2014, 88, 42–54. 10.1016/j.ejmech.2014.07.090. 25081642

[ref51] Wang H.-Y.; Ying Y.-L.; Li Y.; Kraatz H.-B.; Long Y.-T. Nanopore analysis of β-Amyloid peptide aggregation transition induced by small molecules. Anal. Chem. 2011, 83 (5), 1746–1752. 10.1021/ac1029874. 21309531

[ref52] Meyer N.; Abrao-Nemeir I.; Janot J.-M.; Torrent J.; Lepoitevin M.; Balme S. Solid-state and polymer nanopores for protein sensing: A review. Adv. Colloid Interface Sci. 2021, 298, 102561 10.1016/j.cis.2021.102561. 34768135

[ref53] Giamblanco N.; Coglitore D.; Gubbiotti A.; Ma T.; Balanzat E.; Janot J.-M.; Chinappi M.; Balme S. Amyloid growth, inhibition, and real-time enzymatic degradation revealed with single conical nanopore. Anal. Chem. 2018, 90 (21), 12900–12908. 10.1021/acs.analchem.8b03523. 30189140

[ref54] Yusko E. C.; Prangkio P.; Sept D.; Rollings R. C.; Li J.; Mayer M. Single-particle characterization of Aβ oligomers in solution. ACS Nano 2012, 6 (7), 5909–5919. 10.1021/nn300542q. 22686709 PMC3418869

[ref55] Eggenberger O. M.; Leriche G.; Koyanagi T.; Ying C.; Houghtaling J.; Schroeder T. B. H.; Yang J.; Li J.; Hall A.; Mayer M. Fluid surface coatings for solid-state nanopores: comparison of phospholipid bilayers and archaea-inspired lipid monolayers. Nanotechnology 2019, 30 (32), 325504 10.1088/1361-6528/ab19e6. 30991368

[ref56] Eggenberger O. M.; Ying C.; Mayer M. Surface coatings for solid-state nanopores. Nanoscale 2019, 11 (42), 19636–19657. 10.1039/C9NR05367K. 31603455

[ref57] Awasthi S.; Sriboonpeng P.; Ying C.; Houghtaling J.; Shorubalko I.; Marion S.; Davis S. J.; Sola L.; Chiari M.; Radenovic A.; Mayer M. Polymer coatings to minimize protein adsorption in solid-state nanopores. Small Methods 2020, 4 (11), 2000177 10.1002/smtd.202000177.

[ref58] Chen X.; Zhou S.; Wang Y.; Zheng L.; Guan S.; Wang D.; Wang L.; Guan X. Nanopore single-molecule analysis of biomarkers: Providing possible clues to disease diagnosis. TrAC, Trends Anal. Chem. 2023, 162, 117060 10.1016/j.trac.2023.117060. PMC10722900 38106545

[ref59] Acharjee M. C.; Li H.; Rollings R.; Ma B.; Tung S.; Li J. Tau and tubulin protein aggregation characterization by solid-state nanopore method and atomic force microscopy. J. Appl. Phys. 2023, 133 (2), 024701 10.1063/5.0123688.

[ref60] Yusko E. C.; Bruhn B. R.; Eggenberger O. M.; Houghtaling J.; Rollings R. C.; Walsh N. C.; Nandivada S.; Pindrus M.; Hall A. R.; Sept D.; Li J.; Kalonia D. S.; Mayer M. Real-time shape approximation and fingerprinting of single proteins using a nanopore. Nat. Nanotechnol. 2017, 12, 360 10.1038/nnano.2016.267. 27992411

[ref61] Galenkamp N. S.; Biesemans A.; Maglia G. Directional conformer exchange in dihydrofolate reductase revealed by single-molecule nanopore recordings. Nat. Chem. 2020, 12 (5), 481–488. 10.1038/s41557-020-0437-0. 32251371

[ref62] Jiang J.; Li M.-Y.; Wu X.-Y.; Ying Y.-L.; Han H.-X.; Long Y.-T. Protein nanopore reveals the renin–angiotensin system crosstalk with single-amino-acid resolution. Nat. Chem. 2023, 15 (4), 578–586. 10.1038/s41557-023-01139-8. 36805037

[ref63] Ying Y.-L.; Hu Z.-L.; Zhang S.; Qing Y.; Fragasso A.; Maglia G.; Meller A.; Bayley H.; Dekker C.; Long Y.-T. Nanopore-based technologies beyond DNA sequencing. Nat. Nanotechnol. 2022, 17 (11), 1136–1146. 10.1038/s41565-022-01193-2. 36163504

[ref64] Ying Y.-L.; Long Y.-T. Nanopore-based single-biomolecule interfaces: from information to knowledge. J. Am. Chem. Soc. 2019, 141 (40), 15720–15729. 10.1021/jacs.8b11970. 31509414

[ref65] Chen S. W.; Drakulic S.; Deas E.; Ouberai M.; Aprile F. A.; Arranz R.; Ness S.; Roodveldt C.; Guilliams T.; De-Genst E. J.; Klenerman D.; Wood N. W.; Knowles T. P.; Alfonso C.; Rivas G.; Abramov A. Y.; Valpuesta J. M.; Dobson C. M.; Cremades N. Structural characterization of toxic oligomers that are kinetically trapped during alpha-synuclein fibril formation. Proc. Natl. Acad. Sci. U. S. A. 2015, 112 (16), E1994–2003. 10.1073/pnas.1421204112. 25855634 PMC4413268

[ref66] Houghtaling J.; Ying C.; Eggenberger O. M.; Fennouri A.; Nandivada S.; Acharjee M.; Li J.; Hall A. R.; Mayer M. Estimation of shape, volume, and dipole moment of individual proteins freely transiting a synthetic nanopore. ACS Nano 2019, 13 (5), 5231–5242. 10.1021/acsnano.8b09555. 30995394

[ref67] Kumar S. T.; Donzelli S.; Chiki A.; Syed M. M. K.; Lashuel H. A. A simple, versatile and robust centrifugation-based filtration protocol for the isolation and quantification of α-synuclein monomers, oligomers and fibrils: Towards improving experimental reproducibility in α-synuclein research. J. Neurochem. 2020, 153 (1), 103–119. 10.1111/jnc.14955. 31925956 PMC7155127

[ref68] Giehm L.; Svergun D. I.; Otzen D. E.; Vestergaard B. Low-resolution structure of a vesicle disrupting &alpha;-synuclein oligomer that accumulates during fibrillation. Proc. Natl. Acad. Sci. U. S. A. 2011, 108 (8), 3246–3251. 10.1073/pnas.1013225108. 21300904 PMC3044375

[ref69] Näsström T.; Fagerqvist T.; Barbu M.; Karlsson M.; Nikolajeff F.; Kasrayan A.; Ekberg M.; Lannfelt L.; Ingelsson M.; Bergström J. The lipid peroxidation products 4-oxo-2-nonenal and 4-hydroxy-2-nonenal promote the formation of α-synuclein oligomers with distinct biochemical, morphological, and functional properties. Free Radical Biol. Med. 2011, 50 (3), 428–437. 10.1016/j.freeradbiomed.2010.11.027. 21130160

[ref70] Sonn-Segev A.; Belacic K.; Bodrug T.; Young G.; VanderLinden R. T.; Schulman B. A.; Schimpf J.; Friedrich T.; Dip P. V.; Schwartz T. U.; Bauer B.; Peters J.-M.; Struwe W. B.; Benesch J. L. P.; Brown N. G.; Haselbach D.; Kukura P. Quantifying the heterogeneity of macromolecular machines by mass photometry. Nat. Commun. 2020, 11 (1), 1772 10.1038/s41467-020-15642-w. 32286308 PMC7156492

[ref71] Young G.; Hundt N.; Cole D.; Fineberg A.; Andrecka J.; Tyler A.; Olerinyova A.; Ansari A.; Marklund E. G.; Collier M. P.; Chandler S. A.; Tkachenko O.; Allen J.; Crispin M.; Billington N.; Takagi Y.; Sellers J. R.; Eichmann C.; Selenko P.; Frey L.; Riek R.; Galpin M. R.; Struwe W. B.; Benesch J. L. P.; Kukura P. Quantitative mass imaging of single biological macromolecules. Science 2018, 360 (6387), 423–427. 10.1126/science.aar5839. 29700264 PMC6103225

[ref72] Pieri L.; Madiona K.; Melki R. Structural and functional properties of prefibrillar α-synuclein oligomers. Sci. Rep. 2016, 6 (1), 24526 10.1038/srep24526. 27075649 PMC4830946

[ref73] Conway K. A.; Lee S.-J.; Rochet J.-C.; Ding T. T.; Williamson R. E.; Lansbury P. T. Acceleration of oligomerization, not fibrillization, is a shared property of both α-synuclein mutations linked to early-onset Parkinson’s disease: Implications for pathogenesis and therapy. Proc. Natl. Acad. Sci. U. S. A. 2000, 97 (2), 571–576. 10.1073/pnas.97.2.571. 10639120 PMC15371

[ref74] Kim H.-Y.; Cho M.-K.; Kumar A.; Maier E.; Siebenhaar C.; Becker S.; Fernandez C. O.; Lashuel H. A.; Benz R.; Lange A.; Zweckstetter M. Structural properties of pore-forming oligomers of α-synuclein. J. Am. Chem. Soc. 2009, 131 (47), 17482–17489. 10.1021/ja9077599. 19888725

[ref75] Lashuel H. A.; Petre B. M.; Wall J.; Simon M.; Nowak R. J.; Walz T.; Lansbury P. T. α-Synuclein, Especially the Parkinson’s disease-associated mutants, forms pore-like annular and tubular protofibrils. J. Mol. Biol. 2002, 322 (5), 1089–1102. 10.1016/S0022-2836(02)00735-0. 12367530

[ref76] van Diggelen F.; Tepper A. W. J. W.; Apetri M. M.; Otzen D. E. α-Synuclein Oligomers: A Study in Diversity. Israel J. Chem. 2017, 57 (7–8), 699–723. 10.1002/ijch.201600116.

[ref77] Uram J. D.; Ke K.; Mayer M. Noise and bandwidth of current recordings from submicrometer pores and nanopores. ACS Nano 2008, 2 (5), 857–872. 10.1021/nn700322m. 19206482

[ref78] Wanunu M.; Morrison W.; Rabin Y.; Grosberg A. Y.; Meller A. Electrostatic focusing of unlabelled DNA into nanoscale pores using a salt gradient. Nat. Nanotechnol. 2010, 5 (2), 160–165. 10.1038/nnano.2009.379. 20023645 PMC2849735

[ref79] Wang X.; Yu S.; Li F.; Feng T. Detection of α-synuclein oligomers in red blood cells as a potential biomarker of Parkinson’s disease. Neurosci. Lett. 2015, 599, 115–119. 10.1016/j.neulet.2015.05.030. 25998655

[ref80] Li J.; Stein D.; McMullan C.; Branton D.; Aziz M. J.; Golovchenko J. A. Ion-beam sculpting at nanometre length scales. Nature 2001, 412 (6843), 166–169. 10.1038/35084037. 11449268

[ref81] Schneider C. A.; Rasband W. S.; Eliceiri K. W. NIH Image to ImageJ: 25 years of image analysis. Nat. Methods 2012, 9 (7), 671–675. 10.1038/nmeth.2089. 22930834 PMC5554542

