# Supplementary material for: Simultaneous Determination of the Size and Shape of Single α-Synuclein Oligomers in Solution
Source: ACS Nano. 2023 Jun 16;17(13):12325–35. doi: 10.1021/acsnano.3c01393 (PMC10339783; doi:10.1021/acsnano.3c01393)
Supplement: Supplementary file 1 — nn3c01393_si_001.pdf [file nn3c01393_si_001.pdf]

—Supporting Information—

Simultaneous Determination of the Size and Shape of Single α-Synuclein Oligomers in Solution

Saurabh Awasthi[1*], Cuifeng Ying[1], Jiali Li[2], Michael Mayer[1*]

[1] Adolphe Merkle Institute, University of Fribourg, Chemin des Verdiers 4, CH-1700 Fribourg, Switzerland

[2] University of Arkansas, Fayetteville, AR- 72701, United States

*Corresponding Author Email ID: saurabh.awasthi@unifr.ch, michael.mayer@unifr.ch

Supplementary Note 1: *Monomer volume of α-Synuclein*

We determined the volume of αSyn monomers in solution using polymer-coated solid-state nanopores (2M KCl, 10 mM HEPES buffer of pH 7.4). **Supplementary Figure S1C** shows the volume estimates of αSyn monomers based on three independent measurements revealing median volumes ranging from 32 nm^3 to 39 nm^3. These volume estimates are close to a volume of αSyn monomers of ~30 nm^3 as determined by SAXS.[S1] Moreover, we also used the web server 3V[S2] for the assessment of protein volumes using two different three-dimensional structures of αSyn 1XQ8 and 2KKW determined by nuclear magnetic resonance. This approach revealed a volume of ~24 nm^3. To determine the size of αSyn oligomers, we used the mean αSyn monomer volume of 35 nm^3 determined from nanopore-based measurements. The size of αSyn oligomers estimated by

nanopores ranges from dimers to 150-mers, dimers to 175-mers, or trimers to 218-mers, depending on whether we assumed αSyn monomer volumes of 35 nm^3, 30 nm^3, or 24 nm^3, respectively for the calculation. The oligomer size range from dimers to 150-mers that we determined based on an αSyn monomer volume of 35 nm^3 agrees well with previous reports.[S3, S4]

Supplementary Note 2: *Stability of oligomers during nanopore recordings*

In order to test the stability of αSyn oligomers with respect to their size distribution in the recording conditions of nanopores (i. e. 10 mM HEPES (pH 7.4), 2 M KCl), we incubated oligomers in PBS or in recording buffer (containing 2 M KCl) for 1 h at room temperature. Thereafter, we determined the oligomer size distribution by TEM imaging of both preparations. **Supplementary Figure S2** reveals no significant difference in the oligomer sizes for αSyn oligomers incubated for 1 h in PBS or in 2 M KCl. In addition to TEM analysis, we also used single-particle mass analysis by mass photometry to determine the effect of buffer conditions on the size of oligomers. **Supplementary Figure S3** reveals no significant difference in oligomer mass distribution was evident among oligomer samples incubated for 90 min or overnight in PBS or 2 M KCl at room temperature. Note, although we did not observe significant changes in oligomer size distribution in the case of the αSyn oligomers studied here, recording conditions may need to be optimized for other amyloid-forming proteins because the buffer conditions, including ionic strength, pH, type, and valency of salts, presence of other proteins or molecules, as well as stir rate and temperature can all have a significant effect on the aggregation of amyloidogenic proteins.

Supplementary Note 3: *Oligomer size and shape estimation*

We estimated the size and shape using the convolution fitting model, as described in detail in SI-Note 1 of Ref [S4] and SI-Note 2 of Ref [S5]. Only events longer than 150 µs and shorter than 2000 µs were considered for this analysis. Our previous analysis indicates that 150 µs is sufficient time for resolving the shape of freely translocating particles through nanopores at the chosen recording bandwidth of 50 kHz.[S4] To determine size and shape, we generated an experimental cumulative distribution of $\Delta I/I_0$ values from the current trace of each event. This cumulative distribution is then fitted to a convolution-fitting model that considers the geometric effects of ellipsoids in various orientations in an electric field, resulting in shape and volume estimates from each event.[S4, S5] The oligomer size and shape presented in this work are the median values of all estimated volumes and length-to-diameter values from *N* events from each peak in the volume and shape distribution.

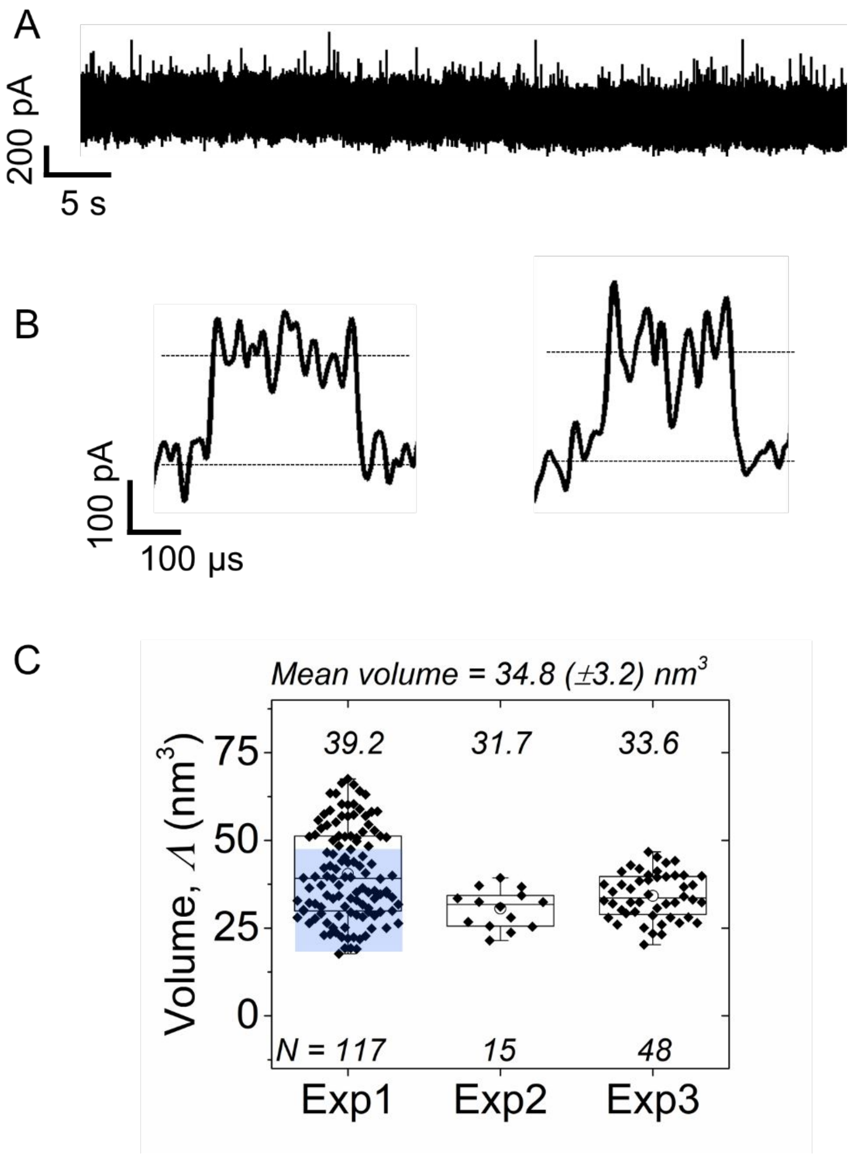

Supplementary Figure S1. Volume of α-Synuclein monomers estimated by resistive pulse sensing. A. Original current trace showing individual translocation events of α-synuclein monomers through a nanopore as upward spikes. **B.** Representative individual translocation events of αSyn monomers. **C.** Volume estimates of αSyn monomer were obtained from three different experiments using small nanopores ranging in diameter between 12 and 16 nm. The average of median volume estimate of αSyn reveals a volume of 34.8±3.2 nm^3 (shown at the top). The diamonds show parameter estimates from intra-event analysis with horizontal lines in the box representing the median and quartile values. The mean value is shown as an open circle for each data set, with the whiskers spanning from the 10th to the 90th percentile. The median values for each data set are shown at the top of the corresponding box plot. The data from Exp. 1 shows that the sample used for this experiment possibly contained not just monomers of αSyn but also a smaller fraction of dimers. Analyzing only the cluster with the smaller volumes (shaded in light blue), reveals a median value of 32.5 nm^3, resulting

in an overall average of median volume of 32.6 (±0.9) nm^3, which is close to the 35 nm^3 that we used throughout this work.

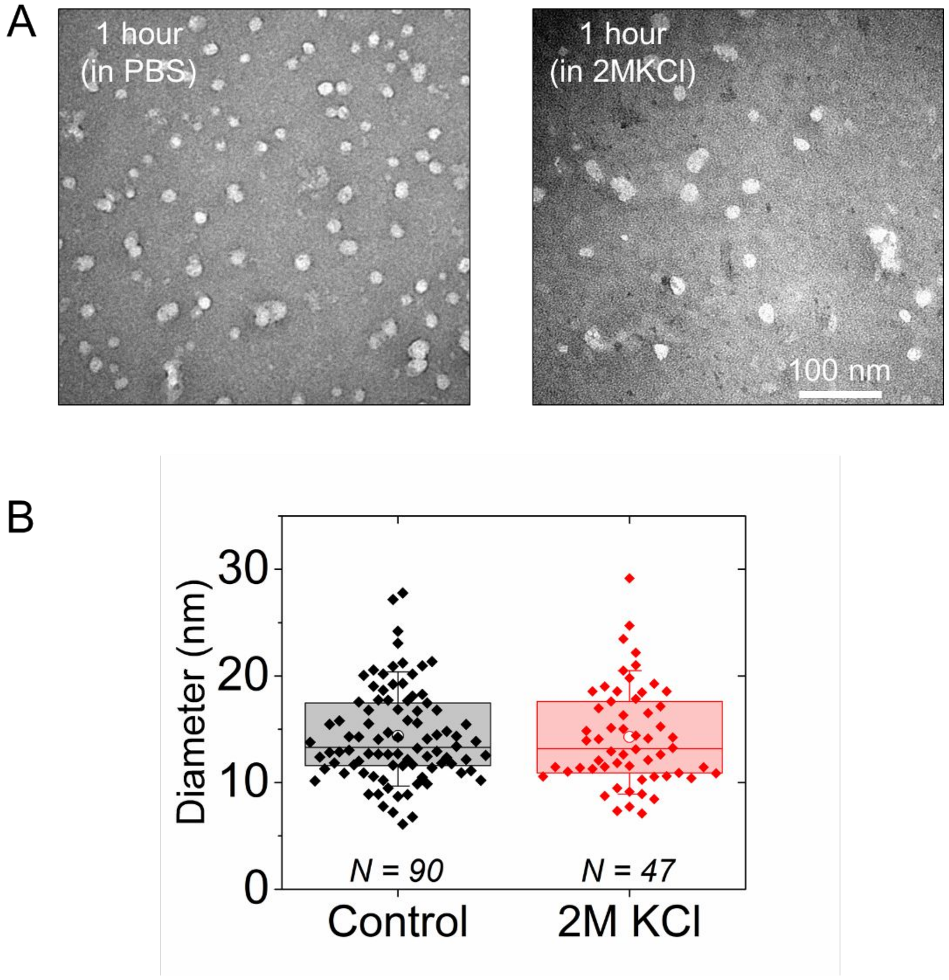

Supplementary Figure S2. Effect of recording buffer on the size of oligomers determined by TEM imaging. **A.** TEM micrographs of αSynuclein oligomers after 1 h of incubation in PBS (control) or in nanopore recording buffer (i.e. 2 M KCl). **B.** Comparative analysis of oligomer size distribution after incubation in PBS buffer or in 2 M KCl. The diamonds show estimates of oligomer diameter from size analysis of single particles with horizontal lines in the box representing the median and quartile values. The mean value is shown as an open circle for each data set, with the whiskers spanning from the 10th to the 90th percentile. No significant change in oligomer sizes occurred within 1 h of incubation in 2 M KCl.

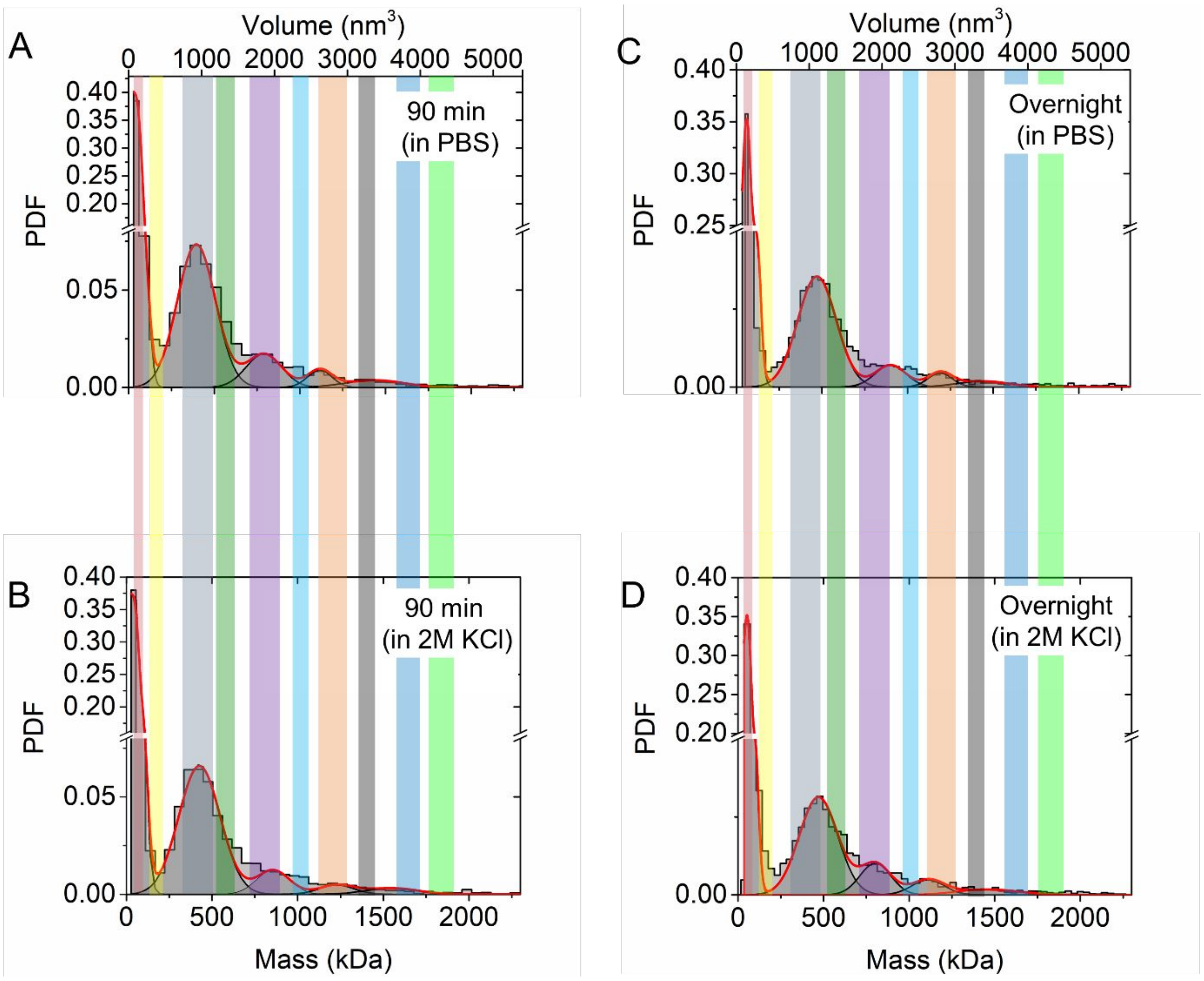

Supplementary Figure S3. Effect of recording buffer on oligomer size distribution determined by mass photometry. A. Size distribution of α-Synuclein oligomers after 90 min incubation in PBS. **B.** Size distribution of α-Synuclein oligomers after 90 min incubation in 2 M KCl. **C.** Size distribution of α-Synuclein oligomers after overnight incubation in PBS. **D.** Size distribution of α-Synuclein oligomers after overnight incubation in 2 M KCl. For comparison, the shaded regions in different colors show the presence/absence of different oligomer populations in different conditions.

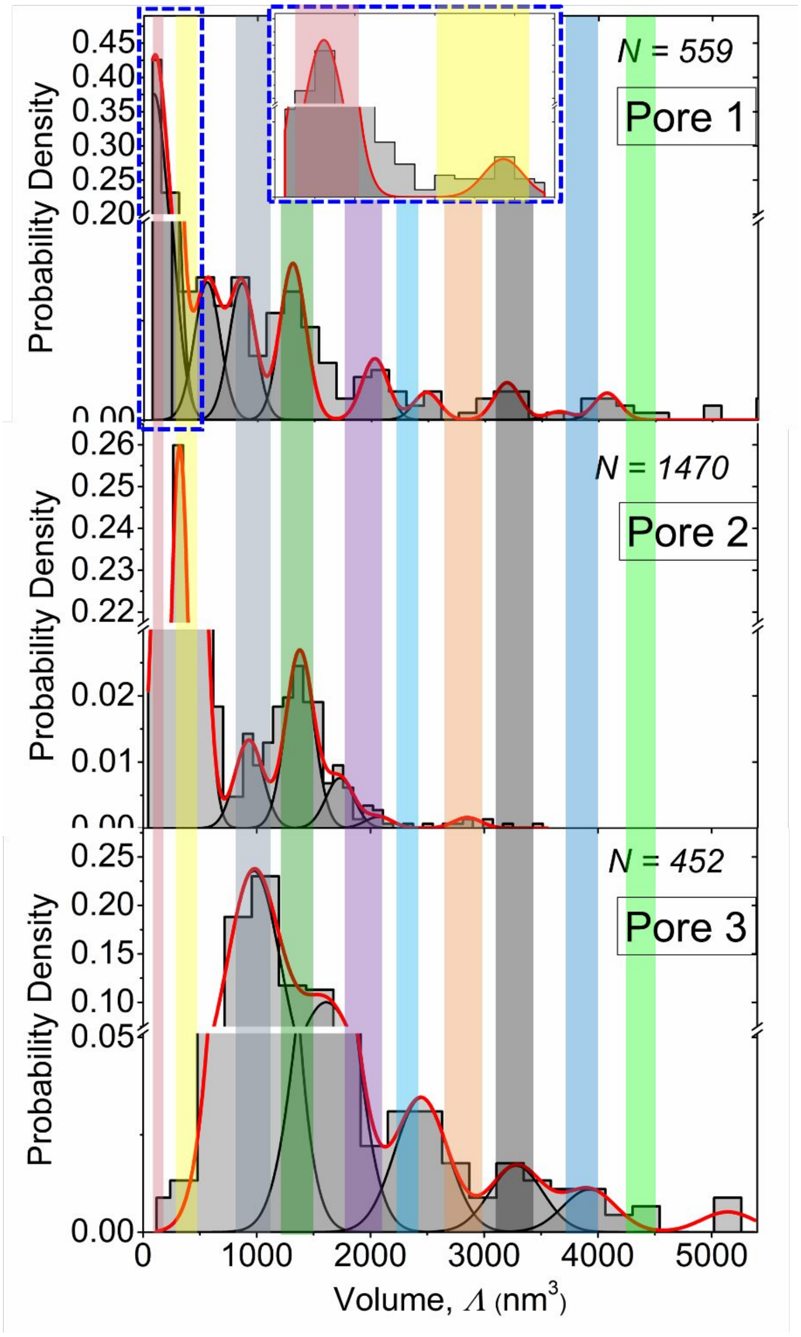

Supplementary Figure S4. Size distributions of α-synuclein oligomers determined using three nanopores with different sizes. A. Size distributions of α-Synuclein oligomers obtained using three different diameters (25 nm, 30 nm, and 56 nm) nanopores. We used three different nanopores with diameters ranging from 25 nm to 56 to capture the wide distribution of αSyn oligomer sizes. The inset with blue dashed outline shows the first two oligomer peaks of the oligomer size distribution determined using pore 1. As mentioned in the Materials and Methods section in the main text, these three nanopores were pre-selected because they resulted in accurate volume estimates of the spherical protein ferritin before they were used for the analysis of αSyn oligomers. The size distribution shown in the main text in Figure 2A is the composite distribution from the three distributions shown here. To identify different-sized oligomer populations in Figure 2A, we used a

multipeak Gaussian fit. Since the fit revealed that the peaks of oligomer populations O^5, O^6, O^7, O^8, O^9, and O^{10} have a similar σ-value, we used a globally-shared σ-value to fit the overall oligomer size distribution in Figure 2A (main text).

Supplementary Table S1. Comparison of oligomer sizes using different methods.

Oligomer size (Number of monomers)	Different methods for oligomer size estimation (Number of monomers)		
	Nanopore	TEM	Mass photometry
O^1 (1-7)	4 (±3)		3 (±1)
O^2 (8-14)	8 (±5)	21 (±16)	
O^3 (15-39)	30 (±9)		35 (±9)
O^4 (40-48)	39 (±9)		
O^5 (49-65)	57 (±8)	47 (±14)	57 (±15)
O^6 (66-76)	69 (±8)		
O^7 (77-87)	82 (±5)	80 (±11)	80 (±6)
O^8 (88-99)	94 (±5)	102 (±11)	103 (±6)
O^9 (100-117)	112 (±7)		
O^{10} (118-127)	122 (±5)	133 (±20)	

Note: Oligomer size in the left-most column refers to the "bin" for grouping oligomer populations with different sizes. We selected 10 bins since we fitted to 10 peaks in the size distribution in Figure 2A in the main text.

Supplementary Table S2. Comparison of different methods used in this work.

Specific features	Technique		
	Nanopore	TEM#	Mass Photometry
Single-particle analysis	Yes	Yes	Yes
Particle size information	Yes	Yes	Yes
Particle shape information	Yes	No	No
Label-free analysis	Yes	No (staining)	Yes
Quantitative analysis	Yes	Yes	Yes
Analysis time	Rapid (~10 min)	Slow (hrs)	Rapid (~10 min)
In solution measurement	Yes	No	Yes
Level of expertise needed	High	High	Moderate
Cost of equipment	Low	Very high	High
Sample preparation	Minimal	Requires chemical fixation or staining of samples	Minimal
Sample volume requirements	Small (5-10 µl)	Small (5-10 µl)	Small (5-10 µl)
Protein/protein aggregate size range	~10 kDa to < 5 MDa (Nanopore diameter can be tuned to adapt for different size proteins and protein aggregates)	<100 kDa (High resolution cryo-EM is required to resolve small size proteins)	40 kDa to 5 MDa[S6]

TEM here refers to dry state transmission electron microscopy.

References

S1. Giehm, L.; Svergun, D. I.; Otzen, D. E.; Vestergaard, B., Low-resolution structure of a vesicle disrupting α-synuclein oligomer that accumulates during fibrillation. *Proc. Natl. Acad. Sci.* **2011,** *108* (8), 3246-3251.

S2. Voss, N. R.; Gerstein, M., 3V: cavity, channel and cleft volume calculator and extractor. *Nucleic Acids Res.* **2010**, 38 (suppl_2), W555-W562.

S2. Cremades, N.; Cohen, S. I. A.; Deas, E.; Abramov, A. Y.; Chen, A. Y.; Orte, A.; Sandal, M.; Clarke, R. W.; Dunne, P.; Aprile, F. A.; Bertoncini, C. W.; Wood, N. W.; Knowles, T. P. J.; Dobson, C. M.; Klenerman, D., Direct observation of the interconversion of normal and toxic forms of α-synuclein. *Cell* **2012,** *149* (5), 1048-1059.

S3. Näsström, T.; Fagerqvist, T.; Barbu, M.; Karlsson, M.; Nikolajeff, F.; Kasrayan, A.; Ekberg, M.; Lannfelt, L.; Ingelsson, M.; Bergström, J., The lipid peroxidation products 4-oxo-2-nonenal and 4-hydroxy-2-nonenal promote the formation of α-synuclein oligomers with distinct biochemical, morphological, and functional properties. *Free Radic. Biol. Med.* **2011,** *50* (3), 428-437.

S4. Yusko, E. C.; Bruhn, B. R.; Eggenberger, O. M.; Houghtaling, J.; Rollings, R. C.; Walsh, N. C.; Nandivada, S.; Pindrus, M.; Hall, A. R.; Sept, D.; Li, J.; Kalonia, D. S.; Mayer, M., Real-time shape approximation and fingerprinting of single proteins using a nanopore. Nat. Nanotech. 2017, 12, 360.

S5. Houghtaling, J.; Ying, C.; Eggenberger, O. M.; Fennouri, A.; Nandivada, S.; Acharjee, M.; Li, J.; Hall, A. R.; Mayer, M., Estimation of Shape, Volume, and Dipole Moment of Individual Proteins Freely Transiting a Synthetic Nanopore. ACS Nano 2019, 13 (5), 5231-5242.

S6. Wu, D., Piszczek, G. Standard protocol for mass photometry experiments. Eur Biophys J. 2021, 50, 403-409.
